# Brazil’s Market for Trading Forest Certificates

**DOI:** 10.1371/journal.pone.0152311

**Published:** 2016-04-06

**Authors:** Britaldo Soares-Filho, Raoni Rajão, Frank Merry, Hermann Rodrigues, Juliana Davis, Letícia Lima, Marcia Macedo, Michael Coe, Arnaldo Carneiro, Leonardo Santiago

**Affiliations:** 1 Centro de Sensoriamento Remoto, Universidade Federal de Minas Gerais, Belo Horizonte, Brazil; 2 Laboratório de Gestão de Serviços Ambientais, Universidade Federal de Minas Gerais, Belo Horizonte, Brazil; 3 Department of Forest Resources and Environmental Conservation, Virginia Tech, Blacksburg, Virginia, United States of America; 4 IRI-THESys, Humboldt-Universität zu Berlin, Unter den Linden 6, Berlin, Germany; 5 Woods Hole Research Center, Falmouth, Massachusetts, United States of America; 6 Agroicone, Av. General Furtado do Nascimento, São Paulo, Brazil; 7 Copenhagen Business School, Solbjerg Pl. 3, Frederiksberg, Denmark; Universidade de Brasilia, BRAZIL

## Abstract

Brazil faces an enormous challenge to implement its revised Forest Code. Despite big losses for the environment, the law introduces new mechanisms to facilitate compliance and foster payment for ecosystem services (PES). The most promising of these is a market for trading forest certificates (CRAs) that allows landowners to offset their restoration obligations by paying for maintaining native vegetation elsewhere. We analyzed the economic potential for the emerging CRA market in Brazil and its implications for PES programs. Results indicate a potential market for trading 4.2 Mha of CRAs with a gross value of US$ 9.2±2.4 billion, with main regional markets forming in the states of Mato Grosso and São Paulo. This would be the largest market for trading forests in the world. Overall, the potential supply of CRAs in Brazilian states exceeds demand, creating an opportunity for additional PES programs to use the CRA market. This expanded market could provide not only monetary incentives to conserve native vegetation, but also environmental co-benefits by fostering PES programs focused on biodiversity, water conservation, and climate regulation. Effective implementation of the Forest Code will be vital to the success of this market and this hurdle brings uncertainty into the market. Long-term commitment, both within Brazil and abroad, will be essential to overcome the many challenges ahead.

## Introduction

Revisions to Brazil’s Forest Code (FC), the principal law regulating forest conservation on private properties, had mixed environmental outcomes [1]. On one hand, the new law reduced reforestation requirements by ≈60%, effectively forgiving the illegal actions of past deforesters. On the other, it maintained most of the original conservation requirements and introduced new mechanisms to facilitate enforcement and compliance. Chief among these are a web-based national land registry (SICAR), mandatory for all 5.4 million rural properties, and the possibility of payments for ecosystem services (PES). The main instrument for the latter is the Environmental Reserve Quotas (Portuguese acronym, CRA) [1], here dubbed “forest certificates”.

Conceived as an offset mechanism, the CRA market allows landowners to lower their costs of compliance by purchasing forest certificates from other properties in lieu of restoring their illegally deforested Legal Reserves (LR)—set aside areas of native vegetation ranging from 80% of the property in the Amazon to 20% in other biomes. The FC specifies that the CRA is a certificate of ownership equivalent to an area of native vegetation [2]. A CRA certificate may be issued for any area of native vegetation exceeding the LR requirement. Yet in the case of small properties―defined as up to four *módulos fiscai*s (MF is a rural unit that varies from 5 ha in densely populated areas to 110 ha in sparsely populated areas like the Amazon), CRA certificates may be issued for the entire area of LR vegetation as well. The law also opens the possibility of issuing CRAs for the expropriation of properties within conservation units and from the joint legal reserves of settlement projects. CRAs must be traded within the same biome and preferably within the same state, but the law also stipulates that CRAs from federally recognized priority conservation areas may be traded across state lines (Figure A in S1 File).

Because the use of CRAs as an offset mechanism eliminates LR areas that would otherwise be restored with native vegetation, it may offer no additionality in terms of carbon sequestration, biodiversity, and other ecosystem services. Even so, the market could bring environmental benefits by increasing the value of native vegetation on private properties. As more rural properties enter SICAR, triggering the 20-year countdown period of the PRA (Portuguese acronym for a landowner‘s official commitment to restore illegally cleared areas (*i*.*e*. the FC debt), interest in the CRA market will likely grow. Advertisements selling forests to compensate LR debts are already appearing in the main Brazilian newspapers, even as states and the federal government engage in a heated debated about regulatory choices for the market.

In a literature review on the CRA and similar forest offset mechanisms, May and colleagues [3] discuss trading possibilities that could harness economic and environmental benefits to the market along with their legal and operational constraints. While some studies have evaluated the potential size of the CRA market across the country (*e*.*g*. [1], [4]), others have focused on the CRA market and similar offset mechanisms in particular Brazilian states (*e*.*g*. [5], [6]). These studies provide a starting point for discussion of the CRA market in Brazil. However, there is little information on how regulatory decisions may influence the economics of the market, including CRA prices and transaction costs, or their environmental implications. Here, we address this gap by analyzing the economic potential of the CRA market and resulting carbon balance (biomass gains or losses) under several regulatory scenarios, discussing its implications for PES programs.

## Materials and Methods

### General approach

We used a suite of models fed with a comprehensive geographic dataset for the entire country of Brazil to analyze the regional CRA markets (Figure B in S1 File). We first calculated the FC surplus and debts using a set of models described in Soares-Filho et al. [1]. These results were then combined with output maps of suitable land for mechanized crops and future agricultural expansion from the OTIMIZAGRO model [7]; data on land titling from the National Institute for Colonization and Agrarian Reform (INCRA); and information on inholdings and priority conservation areas from the Ministry of Environment (MMA) [8] to derive the supplies and demands for CRAs under each modeled scenario. We then associated land prices with CRA supply and demand for each spatial unit, created by intersecting municipality and biome maps. That output, in turn, was fed into a partial equilibrium model to simulate the value and size of regional markets under each modeled scenario. Our contingent valuation indicates that land prices are a proxy for willingness to pay for (WTP) and willingness to accept (WTA) a CRA certificate. To land prices we added fencing and transaction costs obtained through field surveys to estimate regional CRA costs. After running the partial equilibrium model, we calculated the carbon balance under each scenario by superimposing a map of potential forest biomass with the areas traded by the model. Finally, we used OTIMIZAGRO maps to pinpoint areas likely to be deforested in order to illustrate the potential for an expanded CRA market to help avoid deforestation and associated CO_2_ emissions in areas of low land-use opportunity cost. To this end, we built an abatement cost curve using CRA costs from simulated deforestation areas. See methods below.

### 1. Forest code balance and quantity of CRAs

The methodology for calculating the FC surpluses and debts is described in Soares-Filho et al. [1]. To calculate the total supply of CRAs under the law [2], we added the area of forest surplus [1] to that of all native vegetation protected in LRs on small properties. To derive a more realistic estimate of the potential CRA supply, we deducted the areas that are suitable for mechanized agriculture [7, 9] and the percentage of CRAs coming from properties without definitive land titling. To calculate the latter figure, we used municipal information from the agricultural census of the Brazilian Institute for Geography and Statistics (IBGE) [10]. Because IBGE data tends to underestimate the number of properties without land titles (7%), we scaled the municipal data on land titling by using a ratio between IBGE aggregated data and INCRA’s National Rural Registry. The areas of properties to be expropriated within conservation units were supplied by the Instituto Chico Mendes de Conservação da Biodiversidade (Table A in S1 File), while CRAs from settlement projects were estimated by superposing the FC balance map on the map of settlement projects.

### 2. Mapping CRA demand by landowners with high land-use opportunity

To map the potential demand of CRAs from landowners with high land-use opportunity costs and FC debt, we used results from a spatial simulation model, OTIMIZAGRO, an upgraded version of SimAmazonia/SimBrasil [11, 12]. OTIMIZAGRO is a nationwide, spatially-explicit model that simulates land use, land-use change, forestry, deforestation, regrowth, and associated carbon emissions under various scenarios of agricultural land demand and deforestation policies for Brazil [7]. OTIMIZAGRO simulates nine annual crops (*i*.*e*. soy, sugarcane, corn, cotton, wheat, beans, rice, manioc, and tobacco), including single and double cropping; five perennial crops (*i*.*e*. Arabica coffee, Robusta coffee, oranges, bananas, and cocoa); and plantation forests. The model framework, developed using the Dinamica EGO platform [13], is structured in four spatial levels: (i) Brazil's biomes, (ii) IBGE micro-regions, (iii) Brazilian municipalities, and (iv) a raster grid with 25 ha spatial resolution. Concurrent allocation of crops at raster cell resolution is a function of crop aptitude and profitability (Figures C and D in S1 File), calculated using regional selling prices, production and transportation costs [14, 15] (Figures E and F in S1 File). When the available land in a given micro-region (or other specified spatial unit) is insufficient to meet the specified land allocation, OTIMIZAGRO reallocates the distribution of remaining land demands to neighboring regions, creating a spillover effect. Future demand for crops, and deforestation and regrowth rates are exogenous to the model.

Current land use map for Brazil, as of 2012 [7], (Figure G in S1 File) is a composite of datasets including forest remnants from PRODES [16], SOS Mata Atlântica [17], Hansen et al. [18], PROBIO [19] and TerraClass [20]. Urban areas are derived from IBGE census tracts [21]. Initial cropland areas are spatially allocated using soy and sugarcane maps [22] and municipal agricultural data [23] plus maps of crop aptitude [24] and profitability [7] (Figures C and D in S1 File).

The future land use map of agricultural expansion (Figure H in S1 File) is based on projections for 2024 [25] extrapolated to 2030 by using historical trends between 1994 and 2013 [23] (Figure I and Table B in S1 File). Projected annual deforestation rates consist of 2009–2014 averages for the Amazon [16], Cerrado [26], and Atlantic Forest [17], and 2008–2013 averages for the other biomes [18] (Table C in SI File). Since we constrained the model to deforest only in areas of FC surplus [1], the total realized deforestation of 19 Mha is smaller than the projected 21 Mha. The probability of deforestation is a function of spatial determinants, such as distances to roads and previously deforested areas [11]. Forest plantation area by 2030 is specified to address future industry demands [27, 28] and regrowth rates are set to zero in order to identify the areas of future agriculture expansion, where compensation of the forest code debt by purchasing CRA certificates will be more likely to occur. In this way, the model is free to allocate future croplands based solely on crop suitability, regardless of the need to restore LRs. To estimate the CRA demand by landowners with high land-use opportunity, as a last step, we superimposed the land use map of agricultural expansion to 2030 (Figure H in S1 File) on the map of FC debts and surpluses [1].

### 3. Economic valuation of the CRA

We applied a contingent valuation survey [29] to estimate (for those with forest debt) the WTP for a CRA certificate and (for potential sellers) the WTA a CRA price. While it was not possible to obtain a statistically significant sampling of Brazil’s 5.4 million properties, the survey was designed to reduce sampling bias by selecting one state for each of the five regions of the country based on the CRA market potential. Within each selected state, five municipalities were randomly chosen based on their probability proportional to the respective volume of CRAs. For the states of Mato Grosso and Pará, an additional five municipalities were included, given their high potential demand for CRAs. In total, we interviewed 116 landowners, of which 29 come from Pará, 18 from Mato Grosso, 24 from Bahia, 24 from Minas Gerais, and 21 from Paraná.

Contingent valuation is based on the stated preference of economic agents, and as such is sensitive to bias that may arise during interviews. To cope with this, all questionnaires were administered in person by assistant researchers, masters, and PhD students under direct supervision. During data collection, the interviewee had the questionnaire read and explained by the surveyor. The questionnaire was organized in a way that only farmers with forest debt and surplus would be providing answers concerning their willingness to pay or accept CRA titles. In this way, the interviewees provided answers based on a real possibility that they might be trading CRAs [30].

Conventional economic theory prescribes that the present value of the perpetuity of an asset is equal to its selling price. Thus, we asked farmers whether the price of CRA for contracts of different duration would be the productive value of the land (*e*.*g*. rents from cropping and cattle ranching) or the equity value of the land (*e*.*g*. land prices). For 1- to 10-year contracts, most farmers chose land-use rents; for a 30-year contract most farmers indicated land prices as a proxy for WTP for and WTA a CRA (Figure J in S1 File). In this respect, leasing or buying and selling land are viable substitutes for the CRA. The FC establishes that forest debt can also be compensated through land easement, acquisition of private lands inside protected areas or other forested land in the same biome. To cope with the high variance of WTP and WTA as a function of the small sampling size [31], we adopted secondary data on land prices as the basis for a countrywide valuation of CRA titles with 30-year duration.

#### Costs of issuing a CRA certificate

To issue a CRA certificate, landowners must engage in a complex process that involves several steps and incurred costs, as follows: 1) acquiring a copy of the land title from the public notary office and other documents; 2) mapping of the CRA area by a professional surveyor; 3) physical isolation of the CRA area with fencing; 4) evaluation of CRA documentation and in some cases in situ inspection by an environmental agency; 5) notarized registration of the CRA certificate; 6) application for custody of the certificate at an authorized trading market; and 7) payment of capital gain taxes in the event the CRA is traded.

We visited several topographic survey companies in various states of Brazil to estimate mapping costs. To account for cost variability as a function of the property characteristics (*e*.*g*. distance, size, terrain), surveyors were asked to provide maximum and minimum costs they charge for similar services in small (less than 4 MF), medium (4–10 MF) and large properties (more than 10 MF).

Landowners who issue a CRA certificate are legally responsible for conserving the area by isolating it with a fence. Based on field surveys, we estimate the average cost of fencing at US$ 3.56 m^-1^ (1 US$ = 2.15 R$, mean rate of 2013 is used for converting all monetary values). The cost of fencing depends on the shape and size of a CRA parcel in hectares. We assume that those parcels generally approximate a square, fenced on two sides. As a result, the cost of fencing varies from US$ 338/ha (for parcels from 0–20 ha) and US$ 17/ha (for parcels > 2500 ha) (Table D in S1 File).

The costs related to the notary office were calculated based on state-level legislation. We estimated the government fees for field validation and registering the CRA certificate by consulting the state legislation and via phone inquiries with environmental agencies of all 26 Brazilian states and the federal district. Since this specific fee does not exist yet, we used similar services, such as the certification of legal reserves, as a proxy. We adopted a similar procedure to estimate the subscription fee for custody at Cetip (cetip.com.br), one of the two companies authorized by Brazil’s central bank to trade CRA certificates. We also calculated transaction costs for 5-year CRA contracts considering reduced custody costs.

We estimated average costs for small, medium, and large properties (as defined by the mean MF size per state) and then integrated these figures per municipality by using IBGE rural census data [21]. To the sellers' WTA we added mean transaction and fencing costs per CRA parcel size in hectares (Tables D and E in S1 File).

#### Land prices

We used land prices to estimate the basic values of WTP for and WTA a 30-year CRA. This is based on the awareness of landowners of their land values rather than the expectation of rents, as well as the CRA duration period. We used maximum and minimum land prices for three land-use categories, available for Brazilian micro-regions and numerous municipalities [32], to build the maps of land prices. For municipalities where land prices were unavailable, we interpolated from the values of neighboring municipalities to produce a continuous spatial representation (Figure K in S1 File). Since the model includes only the potential demand of CRAs from landowners with high land-use opportunity costs, we used agricultural land prices to represent WTP, and a composite of minimum pastureland and forested land prices to represent the basic value for WTA and its uncertainty bounds. Max = minimum pastureland price. Min = mean(minimum pastureland price, forested land price). Land prices for inholdings come from the database of Bolsa Verde do Rio de Janeiro, www.bvrio.org (Table A in S1 File).

### 4. The partial equilibrium model

**Fig 1:**
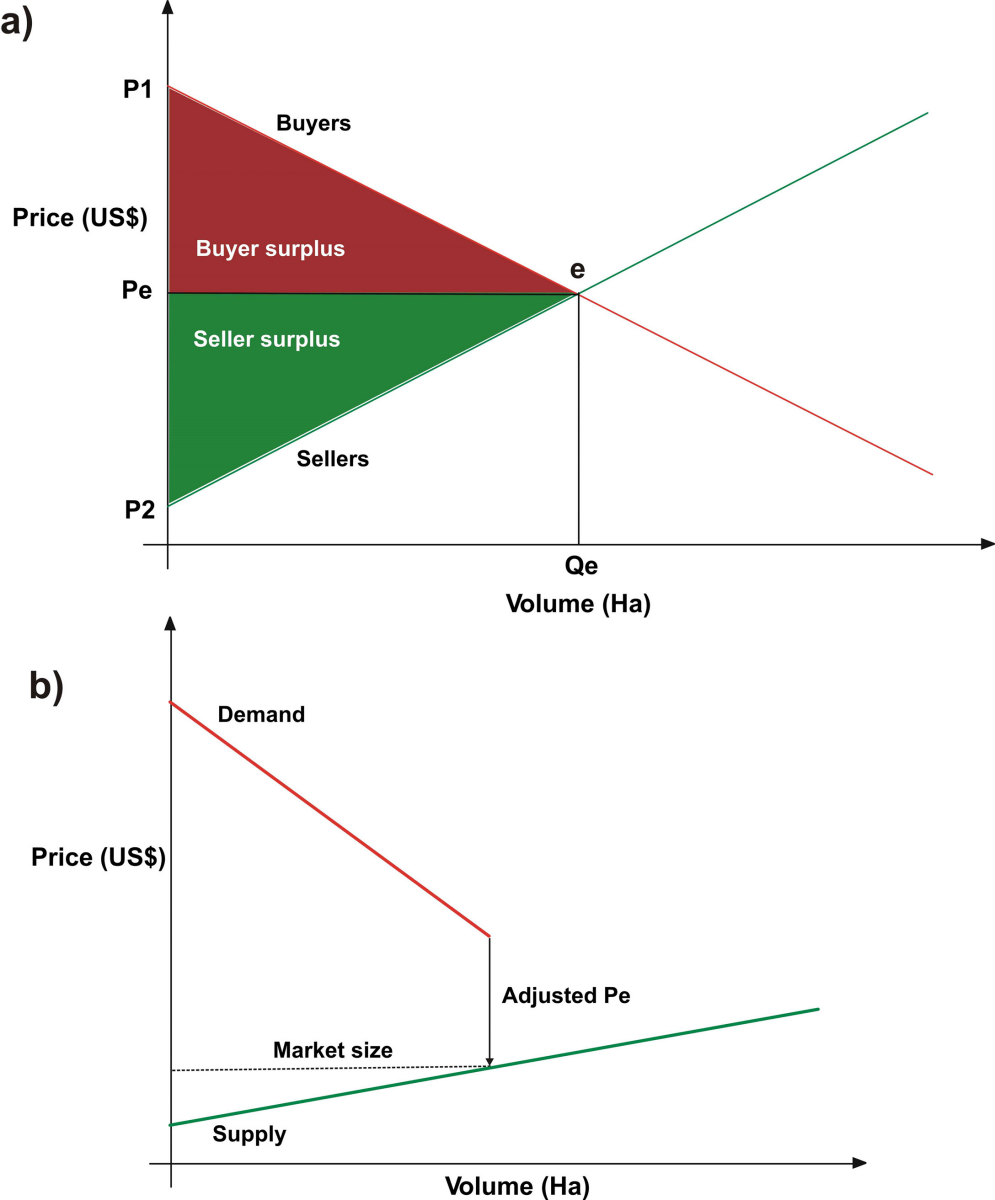
Theoretical partial equilibrium model (a) and the one adapted to assess the CRA market (b). Pe = equilibrium price, Qe = volume at equilibrium.

The model assumes that all buyers and sellers enter the market at the same time; it then calculates the quantity and value of CRAs traded for each unit of biome and state by finding the intersection between the curves of supply and demand expressed in terms of volume (ha) per US$. That is the CRA price and volume per unit of municipality/biome. Theoretically, this point represents the equilibrium price (Pe) of the market [33]; its size = Pe*Qe (Fig 1A). Because this is an imperfect market with predetermined quantities of supply and demand, most of the curves do not cross (Fig 1B). In these cases, Pe is obtained conservatively by projecting the termination of the shorter curve onto the supply curve. The buyers and sellers on the left side of Pe successfully trade CRAs, whereas those on the right side decide not to sell or buy in the face of other land-use alternatives (*i*.*e*. CRA price < WTA, or CRA > WTP). The partial equilibrium model was implemented using Dinamica EGO [13].

#### Regulatory scenarios

Because detailed regulation of the market is pending, we evaluated the economic viability of four possible regulatory scenarios of CRAs issued for a 30-year period: 1) Trading allowable only within the same state and biome, 2) Scenario 1 with the addition of CRAs from priority conservation areas within the same biome, 3) Scenario 1 with the addition of CRAs from conservation units and settlement projects within the same biome, 4) Scenario 3 with CRA trading allowed across state lines.

#### Sensitivity analysis

Given that the CRA market will only be fully realized if the FC is thoroughly enforced, we developed a sensitivity analysis of the market size and value and CRA price as a function of FC compliance by randomly reducing the number of potential CRA buyers by 25%, 50%, and 75%. For each level of compliance, we ran the model 10 times. We also performed a sensitivity analysis of the effect of transaction costs on the CRA cost by running the equilibrium model with CRA contracts of 5-year duration. Following the perception of landholders, Net Present Values (NPV of 5 years, discount rate of 5% a year) of regional land rents from cropping and cattle ranching [34] substitute for WTP and WTA, respectively.

### 5. Avoiding deforestation by purchasing low-cost CRAs

In order to map low-cost CRAs in areas of deforestation pressure, we used the simulated land-use map for 2030. We built an abatement cost curve (accumulated function) of prices of CRAs versus their respective quantities in hectares from simulated deforestation areas, which would become CRA titles instead of being legally deforested. We then selected CRAs from the first half of the curve in order to quantify investments needed to cut deforestation in half by purchasing (as CRA titles) those FC surpluses under deforestation pressure.

### 6. Carbon balance from CRA trading scenarios and avoided CO_2_ emissions by purchasing low-cost CRAs

We estimated the carbon balance between potential sequestration by restoration and reduced emissions from avoided deforestation by superimposing a potential biomass map (see below) on the maps of CRA buyers and sellers. Carbon content is assumed to be 50% of woody biomass [35]. From the difference between CO_2_ contents of areas of buyers and sellers, we subtracted the CO_2_ contents of all CRAs in the Atlantic forest, because the Atlantic Forest Special Protection Regime prohibits deforestation in this biome [36], and the CO_2_ contents of CRAs coming from Legal Reserves and conservation units, since these types of CRAs do not have carbon additionality either. To calculate committed CO_2_ emissions from deforestation, we assumed that 85% of the carbon contained in trees is released to the atmosphere after deforestation [37]. We added 20% to the overall uncertainty to account for the inherent uncertainty in the biomass map. Other sources and sinks of CO_2_ from land use and land use change are not considered.

#### Potential biomass map

The potential biomass map reconstructs the biomass of the original vegetation present in the Brazilian biomes (Figure L in S1 File, S2 File). An extensive literature review on biomass density data was performed considering all the Brazilian biomes and their vegetation types [38]. We collected biomass field measurements reported in national and international scientific papers, technical reports, doctoral dissertations, master's theses, federal and state reports, and biomass inventories. After reviewing 371 sources, 136 were selected as methodological and theoretical support, and 119 used as sources of biomass density data. In addition, several authors were directly consulted for additional information about their methods and unpublished data. Table F in S1 File provides a list of literature sources from authors who provided additional information [39–48].

Research sources were thoroughly screened to select key details of methods used for biomass measurements in the field, vegetation components taken into consideration in biomass calculation, inclusion or exclusion of necromass, informed successional stage, and vegetation type, geographic coordinates of each point of measurement, and accuracy of the coordinates. Biomass values were reported in Mg/ha. As in Fearnside et al. [49], reported vegetation types were reclassified and translated into common names in accordance with the IBGE classification system [38]. Inexact data and outliers were removed. In cases where authors reported one point with geographic coordinates for several measurements in nearby plots, the values were replaced by the average value found. After this process, the selected plots with biomass density totaled 1,045.

For each vegetation type, information on biomass density (Mg/ha) for each component of the vegetation was used to calculate the ratio between components and the total aboveground live biomass. Components whose ratios were calculated are leaves, arborous strata, shrubby strata, herbaceous strata, roots, necromass, palms, and lianas. The calculated ratios were then used as correction factors in order to add biomass values for components not considered by those studies. Each biomass value collected from the literature was adjusted to include all components as in Fearnside et al. [49]; *e*.*g*., data on aboveground live biomass were summed with values for roots and necromass based on ratios from literature values for vegetation types. This process allowed us to compare standardized values, since available values in the literature do not follow a standard procedure. After standardizing plot data to arrive at the total above and below ground biomass, we created a map containing the mean value for each vegetation type (Figure L in S1 File, S2 File).

## Results

### CRA demand and supply

We estimate that 148 Mha of native vegetation on private lands could become CRAs (1 CRA = 1 ha). Of this total, 92 Mha are surplus forest areas―the area of native vegetation exceeding the FC requirements that could be legally deforested―while 55.5 Mha occur within LRs of small properties (Table 1). Large FC surpluses occur principally in the Cerrado and Caatinga biomes across the states of Bahia, Piauí, Mato Grosso, Minas Gerais, Maranhão, and Piauí, while the largest source of CRAs from LRs of small landholders is the Amazon biome, across the states of Amazonas, Pará, Mato Grosso, and Acre (Figures M and N in S1 File). In a scenario where all landowners needing to restore their LRs are willing to buy CRAs and trade is only allowed within the same biome and state, the total supply of CRAs would be enough to meet 87% of the demand of 17–19 Mha of LR debt (Figure O in S1 File). The remaining 13% is located in state/biome units where the regional supply of CRAs is lower than the restoration required, namely in the Atlantic Forest of São Paulo, Paraná, and Mato Grosso do Sul; the Cerrado of São Paulo; and the Amazon region of Tocantins (Figure P in S1 File).

**Table 1:** Composition of supply and demand of CRAs. Values in italic are subtracted from above totals to arrive at new totals (bold letters).

**CRA** (Mha)	**Amazon**	**Caatinga**	**Cerrado**	**Atlantic Forest**	**Pampa**	**Pantanal**	**Total**
Forest Code surplus	12.7	25.8	39.9	3.4	3	7.3	92.1
Legal reserve in properties up to 4 FM	38.3	6.2	7.4	2.8	0.4	0.3	55.5
**Total supply of CRAs**	**51**	**32**	**47.3**	**6.2**	**3.4**	**7.6**	**148**
CRA without land titling	*12*.*8*	*6*.*7*	*9*.*2*	*1*.*1*	*0*.*6*	*1*.*2*	*31*.*7*
**CRA with land titling**	**38.2**	**25.3**	**38.1**	**5.1**	**2.8**	**6.3**	**116**
CRA with high land-use opportunity cost	*1*.*8*	*0*	*7*.*7*	*1*.*1*	*1*.*9*	*0*.*1*	*12*.*7*
**Potential supply of CRA**	**36.4**	**25.3**	**30.4**	**3.9**	**0.9**	**6.3**	**103**
CRA from settlement projects	4.7	1.9	2.9	0.3	0.2	0.4	10.4
CRA from conservation units	14.1	0.3	2	0.6	0	0	16.9
**Expanded supply of CRAs**	**55.2**	**27.4**	**35.3**	**4.8**	**1**	**6.6**	**130**
**CRA demand (Mha)**	**Amazon**	**Caatinga**	**Cerrado**	**Atlantic Forest**	**Pampa**	**Pantanal**	**Total**
LR debt	**8.0**	**0.6**	**4.6**	**5.2**	**0.4**	**0.1**	**18.9**
LR debt in areas of low land-use rents	*6*.*0*	*0*.*6*	*3*.*2*	*4*.*0*	*0*.*3*	*0*.*1*	*14*.*2*
**Effective demand for CRAs**	**2.0**	**0.0**	**1.4**	**1.2**	**0.1**	**0.0**	**4.7**

Realistically, only a fraction of the total supply (Table 1) may end up being traded as CRAs. The requirement that land be properly titled before trading excludes 21% of the total area (32 Mha, Figure Q in S1 File). In addition, 13 Mha of forest surplus occur in areas highly suited for agricultural expansion [9], making these areas unlikely candidates for the CRA market due to their high land-use opportunity cost (Figure R in S1 File). By subtracting these areas, we arrive at a potential supply of 103 Mha of CRAs, located primarily in the Amazon biome (Amazonas and Pará states) and across the Cerrado and Caatinga biomes (Bahia and Piauí states) (Figure S in S1 File). Depending on the regulatory choices adopted, an additional 17 Mha could be supplied from conservation units (Table A in S1 File) and 10 Mha from settlement projects (Figure T in S1 File), totaling 130 Mha (Table 1, Figure U in S1 File).

It is also likely that a large number of landowners will opt to restore their forests on site, rather than enter the CRA market, because most illegally deforested land is currently allocated to cattle ranching. Due to the low rents of ranching (US$ 50–100 ha^-1^year^-1^) [50], it would be more cost effective to simply abandon the illegally deforested areas and allow them to regenerate naturally. Additionally, the high costs of proactive vegetation restoration (US$ 1000–5000 ha^-1^) [51] and unclear regulations about what qualifies as “restored” forest after the allotted 20-year recovery period make it likely that abandonment will be a widespread restoration choice.

**Fig 2:**
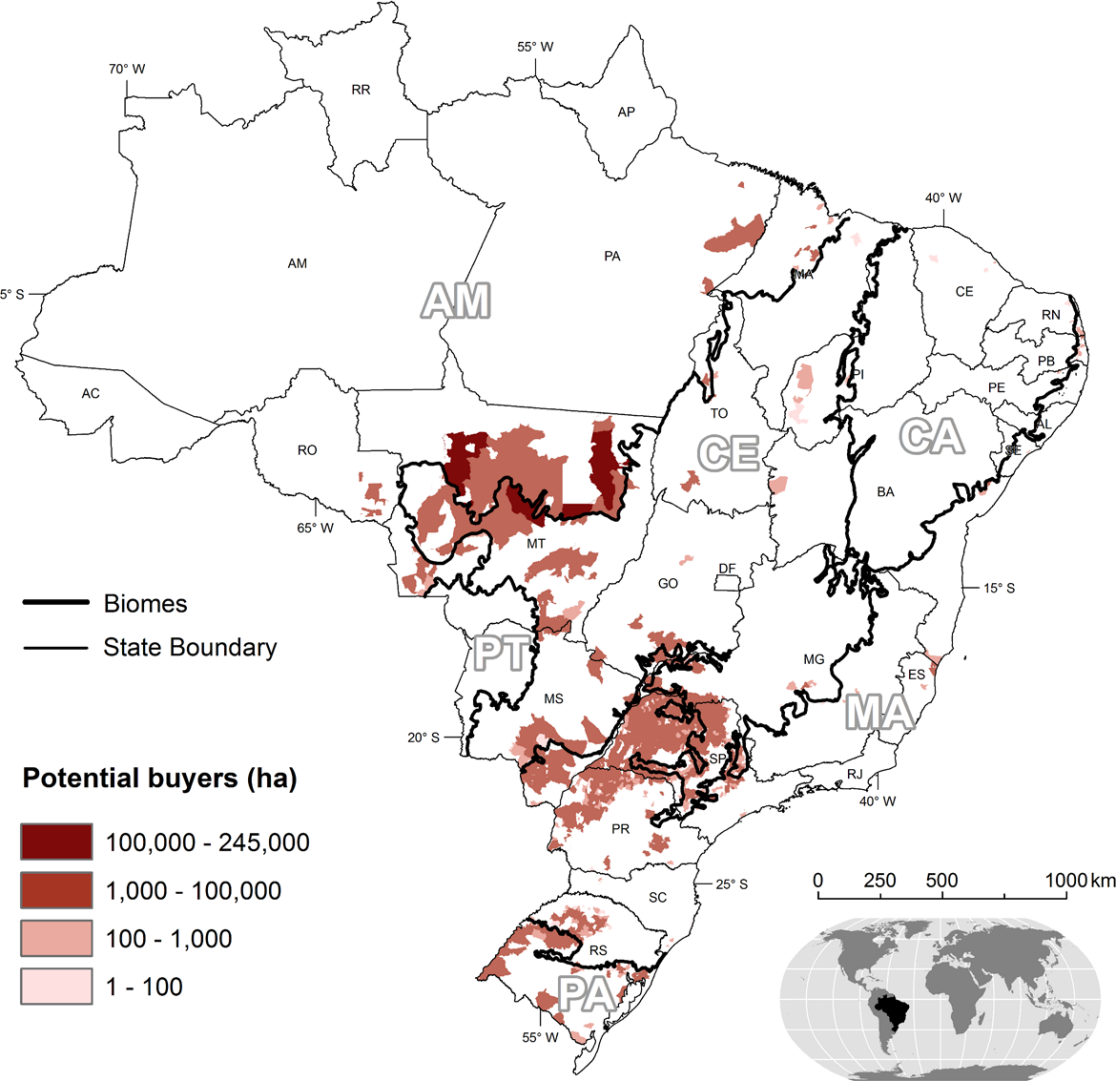
Effective demand for CRA per units of municipality/biome.

Thus, CRAs will be mainly purchased by landowners with high opportunity costs―those, whose lands have restoration obligations but also high profits from mechanized crops (*e*.*g*. sugar cane and soybeans). Moreover, these landowners are more prone to attain compliance, as they have to abide by clean supply chain agreements, such as the soy moratorium [9], and often need access to bank loans that require compliance. The intersection of areas requiring restoration with current and future croplands (Figure H in S1 File) yielded an effective demand of 4.7 Mha for CRAs distributed mainly across the states of Mato Grosso, São Paulo, Paraná, and Mato Grosso do Sul. (Fig 2 and Figure V in S1 File). Note that the effective demand for CRA is a small fraction (25%) of the LR debt (Table 1).

### The CRA market

The analysis of the CRA market is based on a partial equilibrium model that uses a mix of municipal land prices (Figure K in S1 File) to estimate the supply and demand curves, since land prices themselves reflect discounted production returns into infinity―interviews with 116 farmers across five states confirmed the accuracy of this proxy (Figure J in S1 File). The choice to enter the market is thus a function of relative land prices. An individual with a compensation requirement (buyer) will compare the cost of the CRA to the land use rents that would be forsaken and elect to buy a CRA if it costs less than his or her land price. Conversely, an individual will enter the market to sell if the CRA price is higher than his or her land price. Because both buyers and sellers decide based on potential financial returns, land prices are an effective means of estimating participation in the CRA market. However, since there are some significant barriers to entry into the selling market, two additional steps are required: the addition of regional transaction costs (Table E in S1 File), and the cost of fencing needed to isolate the CRA area (Table D in S1 File).

**Fig 3:**
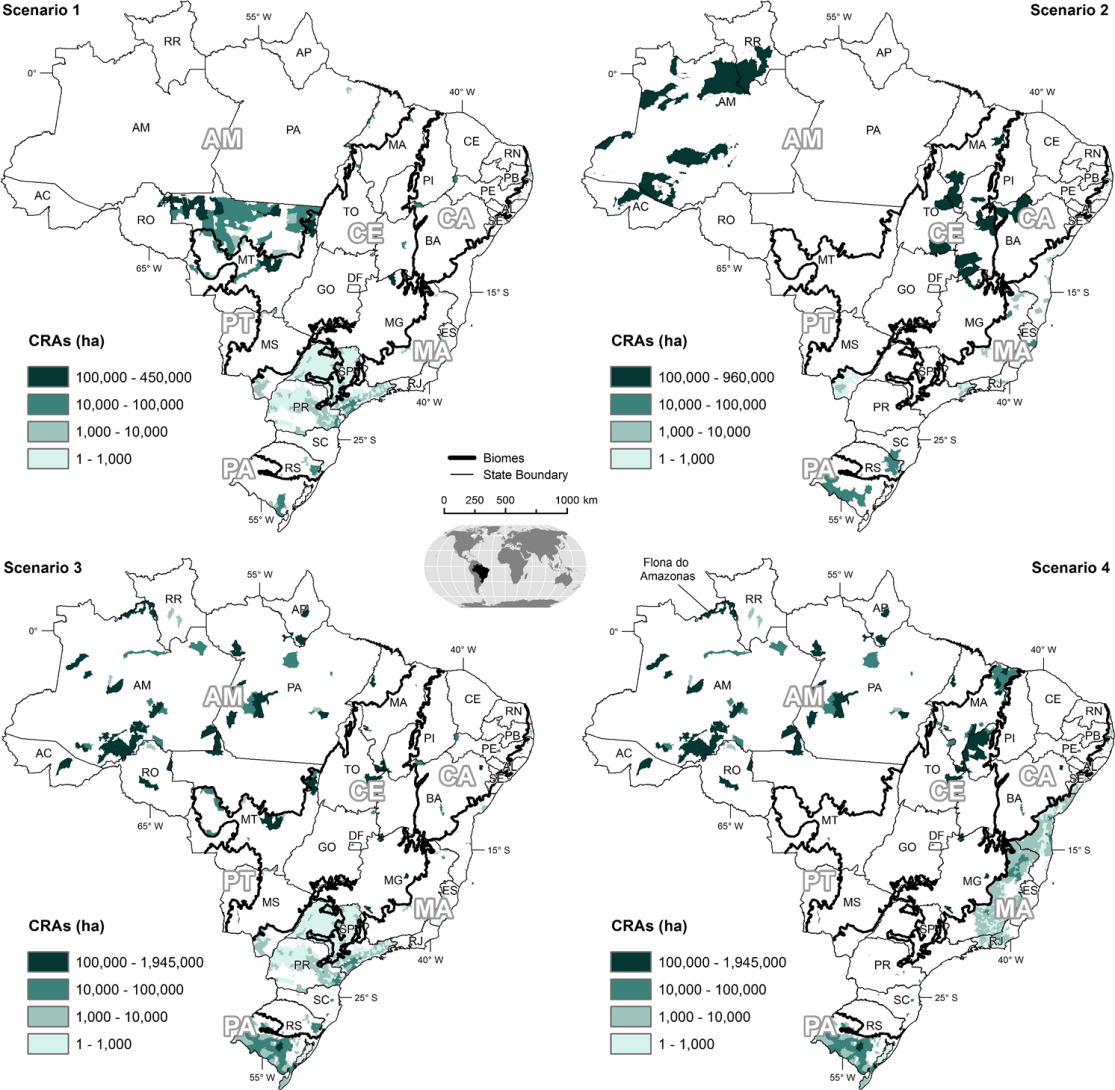
Locations of CRAs traded under the regulatory scenarios per municipality/biome.

Model results show that the more constrained the market, the bigger is its economic potential, and the smaller the net loss of CO_2_ between potential sequestration by restoration that would be offset and emissions from avoided legal deforestation by trading CRAs (Table 2). A negative CO_2_ balance is thus due to swapping LR debts with CRA certificates from areas already protected by the FC, such as LRs from small landholders or inholdings. A larger supply of CRAs from a wider geographic area (Fig 3) depresses the CRA price and hence the total market value despite larger trading volumes (Table 2). In this respect, trading CRAs from inholdings is particularly damaging, given that the provision of CRAs from a single park (*e*.*g*. *Flona do Amazonas*) could satisfy virtually all of the demand (1.97 Mha of CRAs) in the Amazon biome (Table A in S1 File and S2 File).

**Table 2:** Summary figures for the CRA market under regulatory scenarios. Negative value means net CO_2_ loss.

n.	Regulatory Scenario	Mean CRA price (10^3^ US$/ha)	Area offset (10^6^ Ha)	Market value (10^9^ US$)	CO_2_ balance(10^9^ tons)
1	CRAs traded within the same biome and state	2.2±.0.6	4.2±0.0	9.2±2.4	-0.9±0.3
2	CRAs traded within the same biome and state plus CRAs from conservation priority areas across states	1.7±0.4	4.7±0.0	8.1±2.0	-1.0±0.3
3	CRAs traded within the same biome and state plus CRAs from conservation units and settlement projects across states	1.1±0.2	4.7±0.0	5.9±0.7	-2.2±0.4
4	CRAs traded within the same biome but across states plus CRAs from conservation units and settlement projects across states	0.9±0.1	4.7±0.0	4.9±0.7	-2.2±0.4

The best market situation ensues from regulatory scenario 1, totaling 4.2 Mha of CRAs with a gross value of US$ 9.2±2.4 billion. This is relevant given that some Brazilian states have already passed regulations barring trading beyond their borders, whereas others are proposing to open the market for CRAs from other states. Under this scenario, the Amazon and Cerrado biomes in Mato Grosso are by far the largest markets with trading volumes of 1.9 and 0.9 Mha, respectively, and corresponding CRA prices averaging US$ 1,440±300 and US$ 1,430±400 (Table G in S1 File). The Atlantic Forest in São Paulo comes in third with a volume of 0.5 Mha. Nevertheless, it surpasses the total value of Mato Grosso Cerrado because of higher CRA prices, which average US$ 5,520±1,770. CRA markets are also viable in the states of Paraná, Rio Grande do Sul, and Mato Grosso do Sul. Although the model indicates smaller volumes of CRA transactions in other states, their markets may not materialize due to a significant imbalance between supply and demand (Figure W in S1 File).

A fully-fledged CRA market will only materialize if the FC becomes thoroughly enforced. In this respect, the sensitivity analysis we performed indicates that the market size and value decrease at the same rate as that of the level of FC compliance (Figure X in S1 File). Nevertheless, CRA prices decrease only slightly (by 9% at 25% FC compliance) since there is a minimum price CRA holders are willing to sell for.

Transaction and fencing costs have a substantial impact on the final price of the CRAs. On average, transaction costs for 30-year contracts represent 7.5% of the total price, while fencing costs reach 13.6%. For contracts of shorter duration, these upfront investments become even more substantial. A sensitivity analysis with 5-year CRA contracts traded within the same state and biome shows that market size and value would plunge to 1.6 Mha and US$ 1.3±0.1 billion, respectively. This is due to the high ratio of transaction and fencing costs to WTA values (5-year NPV of land-use rents), which in this case reach 16% and 33% of the total CRA price, respectively (Tables D and E in S1 File).

## Discussion

Despite the risk that oversupply may flood many of the regional CRA markets, our results suggest that Brazil’s CRA market could become the largest market for trading forest certificates in the world, yielding 3 times the value and 22 times the land area as that traded by all biodiversity offsets worldwide in 2011 [52]. Even so the CRA market is finite and will come to an end as it self-consumes. Furthermore, CRA trading will not necessarily prevent legal deforestation, particularly in the Cerrado where there are 40 Mha of FC surplus [1], given that most CRAs could come from areas of low deforestation pressure or already protected by FC.

Because high upfront investments are required to obtain the CRA certificate, and these costs vary as a function of the total number of CRAs traded jointly (Tables D and E in S1 File), it is unlikely that small and undercapitalized landholders will supply the majority of CRAs to the market. For the same reason, the view that CRAs from marginal lands will be the first to enter the market [3] may not materialize. Certainty that CRA trading will pay off initial investments could underpin the landowner's decision, making it likely that prior information about the market―such as that provided here―will be more decisive in determining early entry into the market than relative land prices.

**Fig 4:**
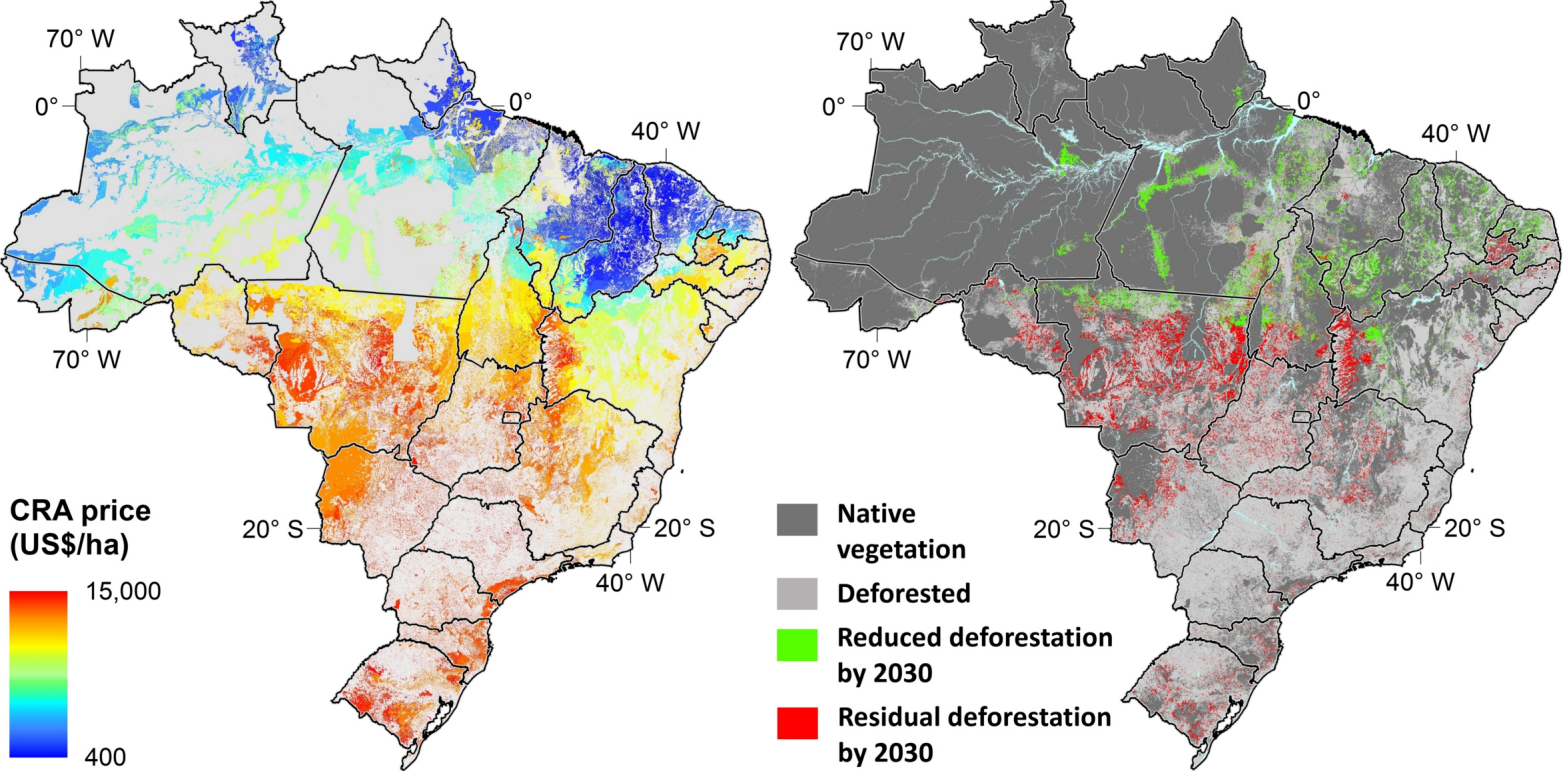
Reduced legal deforestation by purchasing CRAs. (**A**) Prices of CRA from private properties, (**B**) simulated legal deforestation by 2030 and areas where low-cost CRA could be purchased to reduce deforestation by half (areas are enlarged for better visualization).

To overcome the current limitations of the CRA market, its trading platform could be adapted to serve as a common financial mechanism for a wide variety of PES programs. We denote this concept as X-CRA, indicating that the environmental benefits of CRA trading could be multiplied beyond the compensation of the FC obligation. The advantages are many. In contrast to reference levels for rewarding reduced emissions from deforestation [53], uncertainty of CRA is low because it is measured in hectares and thus can be monitored, reported, and verified (MRV) using SICAR. In addition, transactions and sunk costs are much lower for both landowners and the MRV system. Oversupply would not be a problem given that thematic PES programs would target specific subsets of CRAs. Adherence to the market will depend greatly on the demand for a particular ecosystem service, since the supply is essentially elastic; one need only abandon and isolate an area and await vegetation regrowth to obtain a CRA certificate. PES programs aimed at other benefits, such as biodiversity, water resources and forest carbon, would need to map regions of interest in order to identify, foment, and purchase CRAs that match their conservation criteria. To illustrate the potential of linked PES programs, we simulated current land use trends to 2030, estimating that an investment of US$ 8.4±2.0 billion to purchase low-cost CRAs (Fig 4 and Figure Y in S1 File) could cut legal deforestation (19 Mha) in half. This initiative would reduce CO_2_ emissions by as much as 3.8±0.8 billion tons, not to mention the multiple environmental benefits provided [54].

Actions like this will promote other uses for the CRA market. For example, biodiversity offsets [55] and protected area programs [56] could capitalize on the CRA market to build mosaics of set-aside conservation areas on private properties. This has the advantage of much lower fixed costs than those of public conservation units (or acquisition costs, especially in biomes where public land is unavailable), given that private landowners will be the ones chiefly responsible for maintaining the areas. Still, market regulation is needed to facilitate transactions and detail disbursement mechanisms―annuities rather than upfront payment―to ensure engagement of landowners over the long run.

The expanded use of the CRA market will be critical for its broad success. In effect, all of this will depend on the full implementation of the FC. By May 2015, SICAR reportedly had made tremendous headway, registering 1.5 million properties covering 212 Mha [57], but several challenges remain. The SICAR registry (*i*.*e*. CAR) is a self-reporting system and hence needs validation and monitoring. Few states have the capacity to effectively monitor CAR and, even in those that do, the CAR has proved ineffective in curbing deforestation within registered properties [58]. Overall, there is a lack of human and technical resources to carry out field or visual validation. In turn, automatic validation is still flawed due to the absence of accurate cartographic data. To improve the CAR, Brazil needs to carry out systematic mapping of the entire country at a scale ≥ 1: 10,000. Without this information it would be fruitless to develop an automatic validation method [3], let alone a monitoring system necessary to determine compliance after registration. Also, there is a need to couple the CAR to land tenure systems in order to verify property boundaries. All these efforts will require substantial investments that may not be attainable or in line with Brazil’s pressing socioeconomic needs. Finally, contradictory interpretations of the FC law, stemming from conflicting state and federal legislation and lack of further FC regulation, may hinder enforcement, encouraging landowners to disregard the law, hence undermining the CRA market.

It is critical that federal and state governments rapidly advance implementation and regulation of the FC, which is rather uncommon in Brazilian public policies. Moreover, the soybean and beef moratoria (currently in place in the Amazon) ignore suppliers with FC debt, as they are concerned only with recent clearings [9, 59]. The estimated demand for CRAs will materialize only if farmers and ranchers are pushed by aggressive governmental interventions (*e*.*g*. fines, credit or land transaction restrictions) and by more stringent voluntary supply chain agreements.

If successful, the CRA will emerge as a unique mechanism to increase forest value that could further a comprehensive market for PES. The multiplication of PES using the CRA market could provide important ecosystem services, such as protecting the watersheds around reservoirs that supply Brazil’s large urban centers with freshwater [60]. This has become crucial to cope with increasingly frequent droughts, especially in Southeastern Brazil [61]. More importantly, an expanded market of forest certificates (X-CRA) could be integrated into the country’s national strategy for Reducing Emissions from Deforestation and Forest Degradation (REDD+) as the Green Climate Fund becomes operational. This will be central to help Brazil achieve its Intended Nationally Determined Contributions (INDC) to climate change mitigation [62]. The development of these prospects will certainly demand substantial research, offering opportunity for new studies on the application of the CRA market infrastructure to PES.

In sum, an expanded CRA market could be an excellent opportunity to reconcile conservation with agricultural production across the country. Agriculture in Brazil and elsewhere would benefit from climate stability and various other ecosystem services provided by the country’s far-reaching native ecosystems. Long-term commitment, both within Brazil and abroad, will be essential to overcome the many challenges ahead.

## Supporting Information

S1 File All SI figures and SI tables. (PDF)

S2 File Biomass map and list of inholdings. (ZIP)
