# Correction: Brazil's Market for Trading Forest Certificates

**DOI:** 10.1371/journal.pone.0157203

**Published:** 2016-06-03

**Authors:** The PLOS ONE Staff

There is an error in the fourth sentence of the second paragraph of the Introduction. The publisher apologizes for the error. The correct sentence is: Yet in the case of small properties—defined as up to four módulos fiscais (MF is a rural unit that varies from 5 ha in densely populated areas to 110 ha in sparsely populated areas like the Amazon), CRA certificates may be issued for the entire area of LR vegetation as well.

There is an error in the last sentence of the second paragraph of the Discussion. The publisher apologizes for the error. The correct sentence is: Certainty that CRA trading will pay off initial investments could underpin the landowner's decision, making it likely that prior information about the market—such as that provided here—will be more decisive in determining early entry into the market than relative land prices.

There is an error in the last sentence of the fourth paragraph of the Discussion. The publisher apologizes for the error. The correct sentence is: Still, market regulation is needed to facilitate transactions and detail disbursement mechanisms—annuities rather than upfront payment—to ensure engagement of landowners over the long run.

There is an error in the second sentence of the “CRA demand and supply” subsection of the Results. The publisher apologizes for the error. The correct sentence is: Of this total, 92 Mha are surplus forest areas—the area of native vegetation exceeding the FC requirements that could be legally deforested—while 55.5 Mha occur within LRs of small properties (Table 1).

There is an error in the first sentence of the subsection “The CRA market” of the Results. The publisher apologizes for the error. The correct sentence is: The analysis of the CRA market is based on a partial equilibrium model that uses a mix of municipal land prices (Figure K in S1 File) to estimate the supply and demand curves, since land prices themselves reflect discounted production returns into infinity—interviews with 116 farmers across five states confirmed the accuracy of this proxy (Figure J in S1 File).

The following information is missing from the Funding section: This work was supported by the Climate and Land Use Alliance, Ministério do Meio Ambiente and Deutsche Gesellschaft für Internationale Zusammenarbeit via Project TEEB Regional-Local, Conselho Nacional de Desenvolvimento Científico e Tecnológico, Fundação de Amparo à Pesquisa do Estado de Minas Gerais, Servamb, and the Gordon and Betty Moore Foundation.

The following information is missing from the Acknowledgments: We would also like to thank Ana Luiza Champloni and Aloísio de Melo for ideas.
