# Supplementary material for: Brazil’s Market for Trading Forest Certificates
Source: PLoS One. 2016 Apr 6;11(4):e0152311. doi: 10.1371/journal.pone.0152311 (PMC4822866; doi:10.1371/journal.pone.0152311)
Supplement: S1 File — (PDF) [file pone.0152311.s001.pdf]

S1 File

Brazil's market for trading forest certificates

Britaldo Soares-Filho, Raoni Rajão, Frank Merry, Hermann Rodrigues, Juliana Davis, Letícia Lima, Marcia Macedo, Michael Coe, Arnaldo Carneiro, Leonardo Santiago

SI Figures

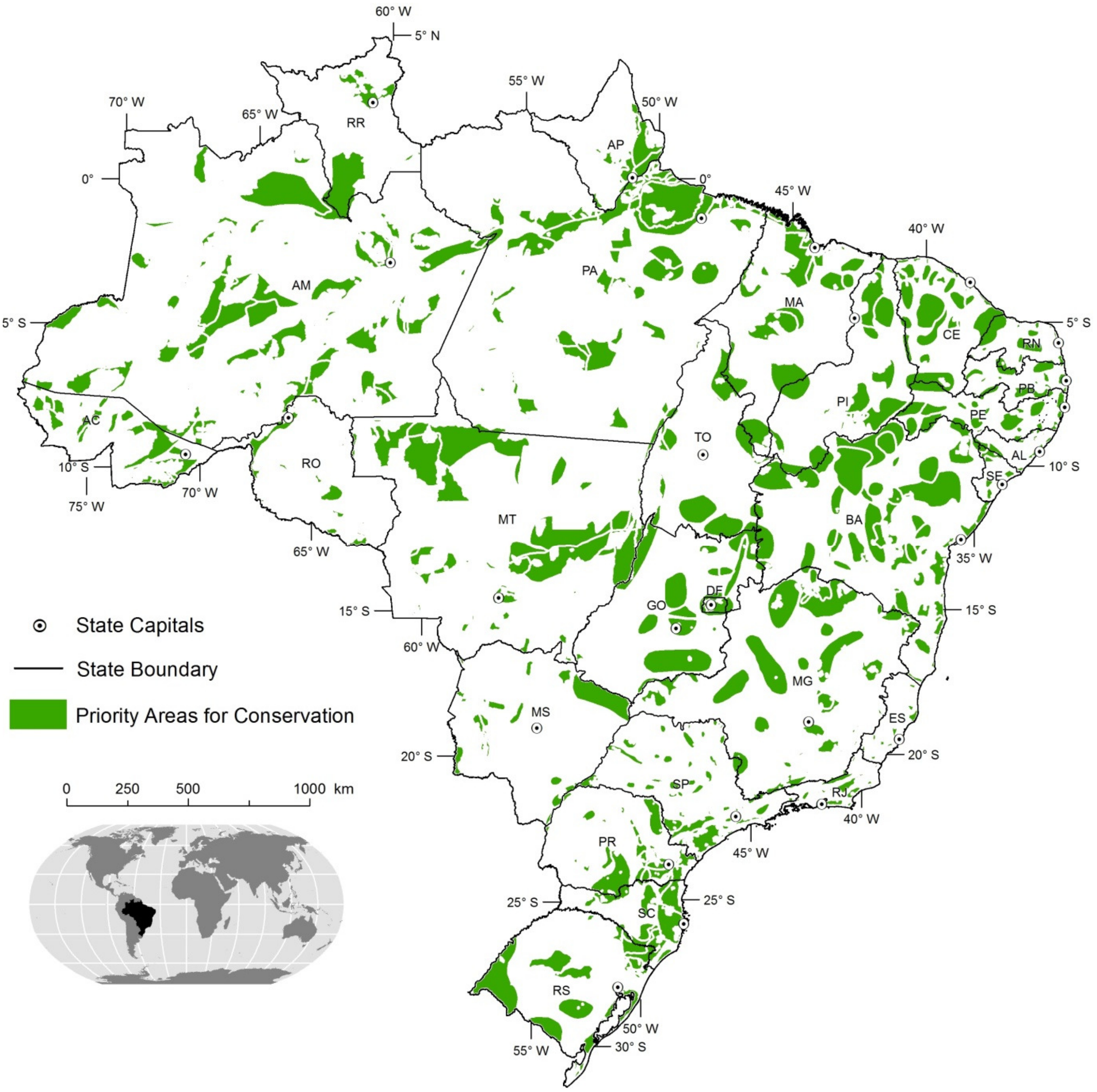

Fig A. Priority areas for conservation outside of protected areas [8].

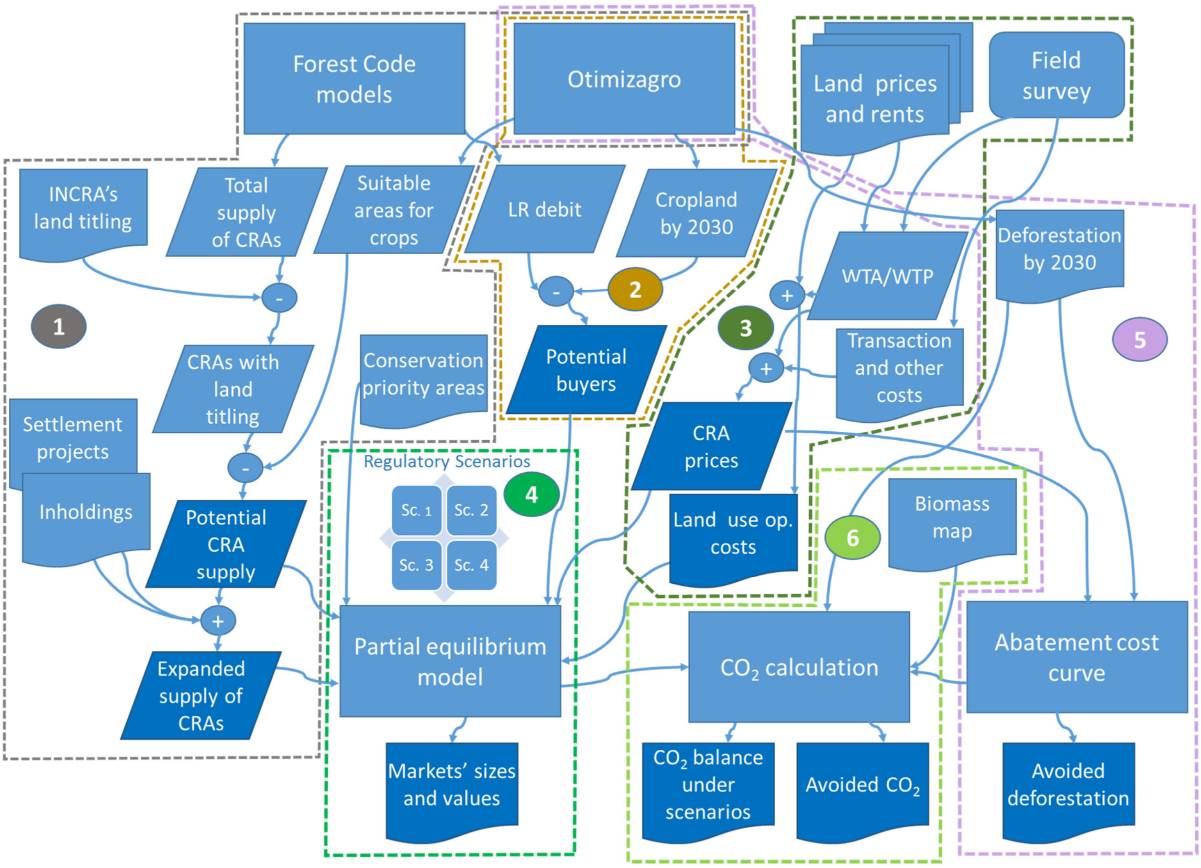

Fig B. Modeling flowchart with the main results highlighted in dark blue. Dashed lines envelop models and processes described in the respective method sections together with outputs.

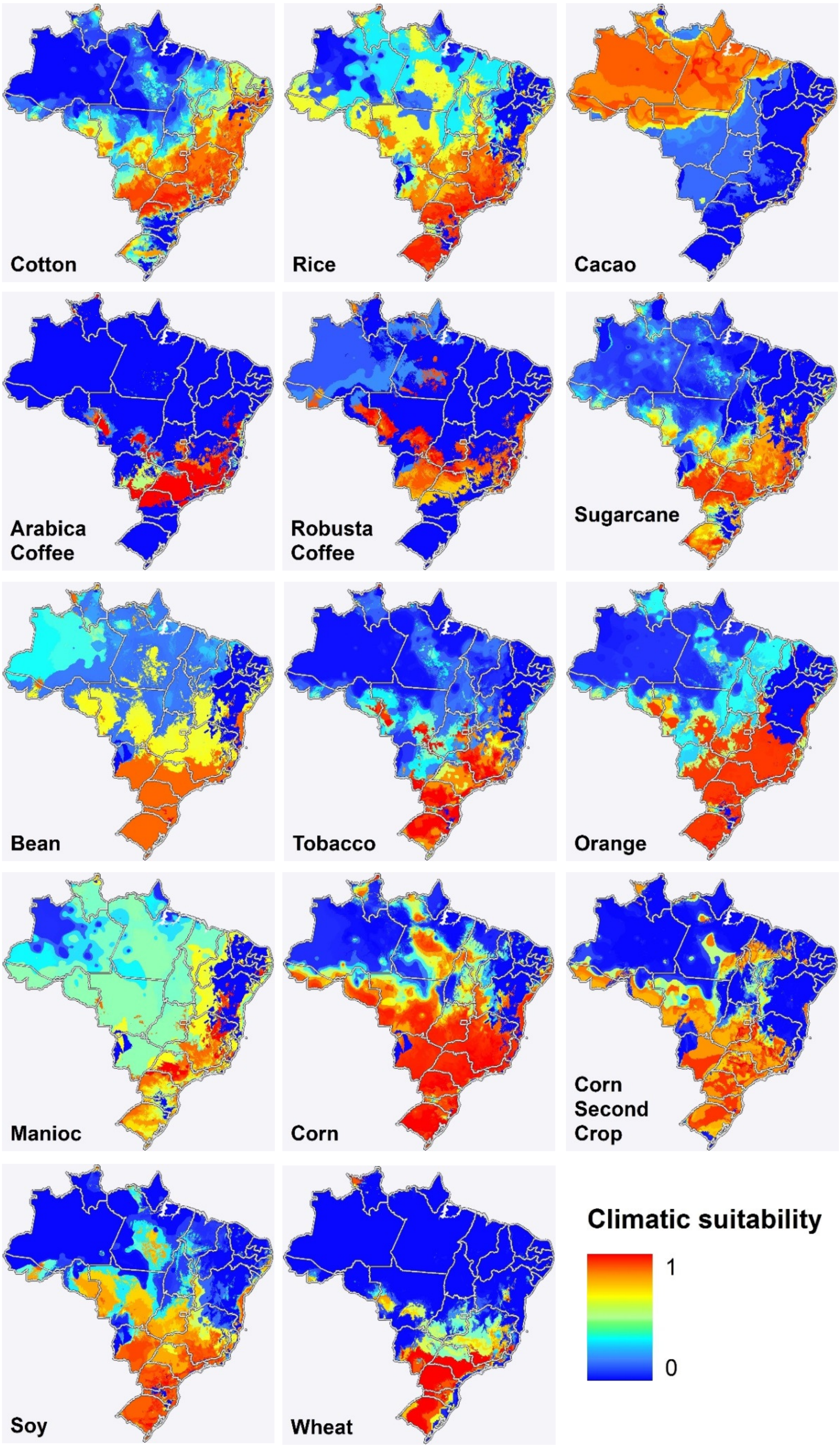

Fig C. Climatic suitability maps [24].

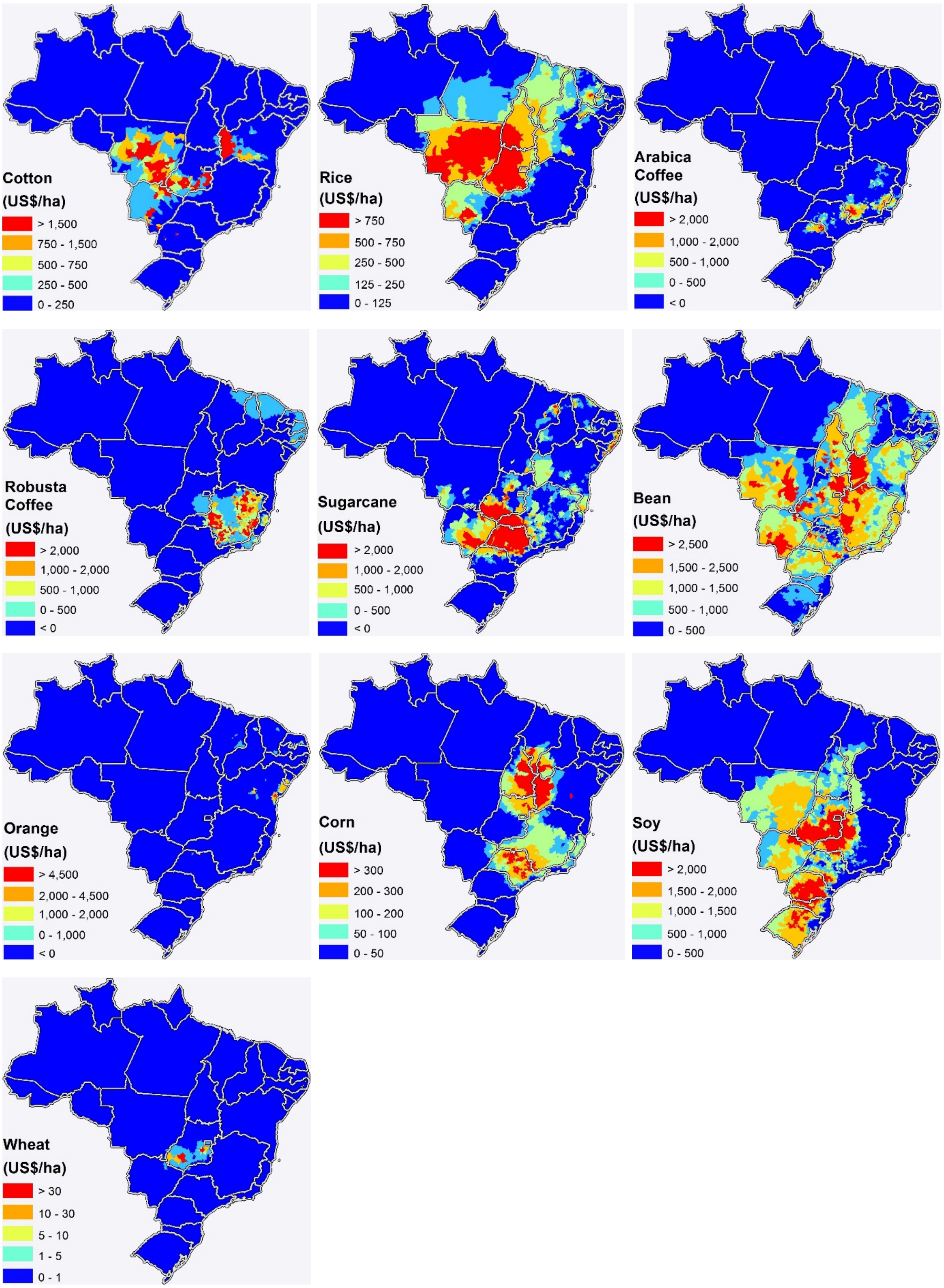

Fig D. Rents (Net Present Value for 30 years, discount rate of 5% a year) for major crops between 2009 and 2010 [7].

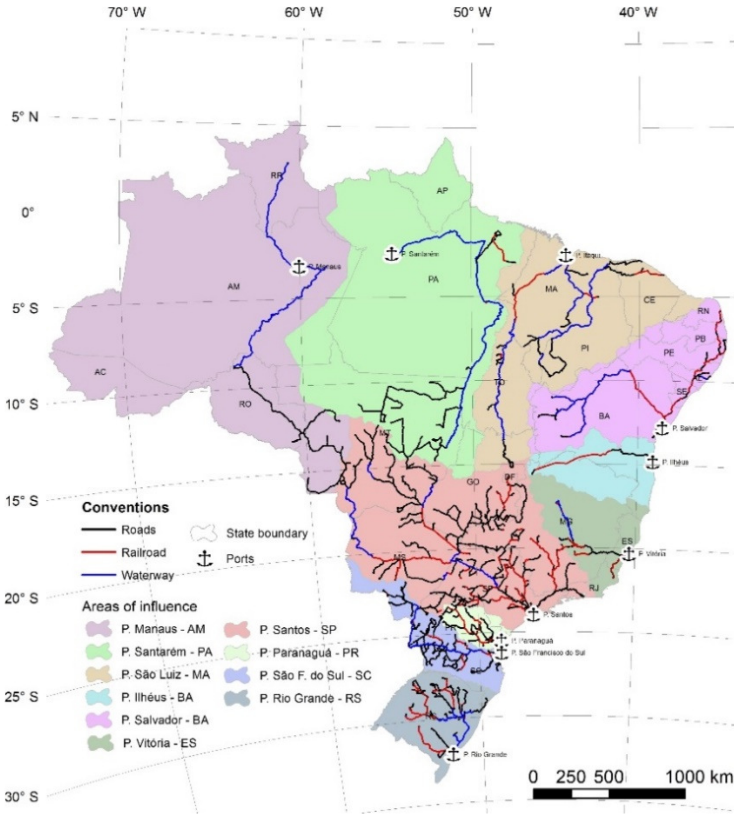

Fig E. Current transportation network with areas of influence of major exportation ports [15].

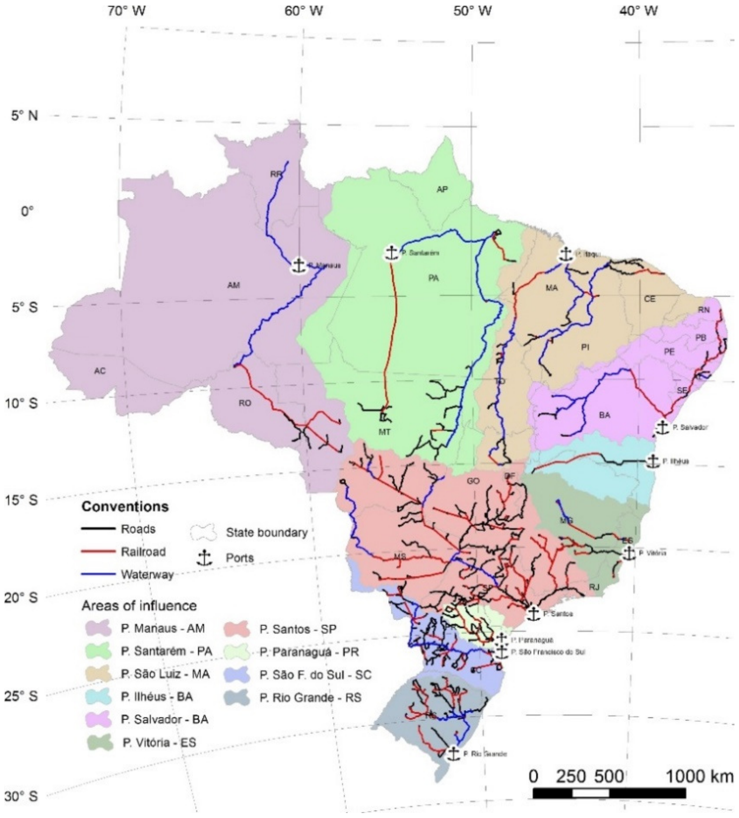

Fig F. Transportation network planned by "*Plano Nacional de Logística e Transportes*", PNLT (http://www.transportes.gov.br/images/2014/11/PNLT/2011.pdf) with areas of influence major exportation ports [15].

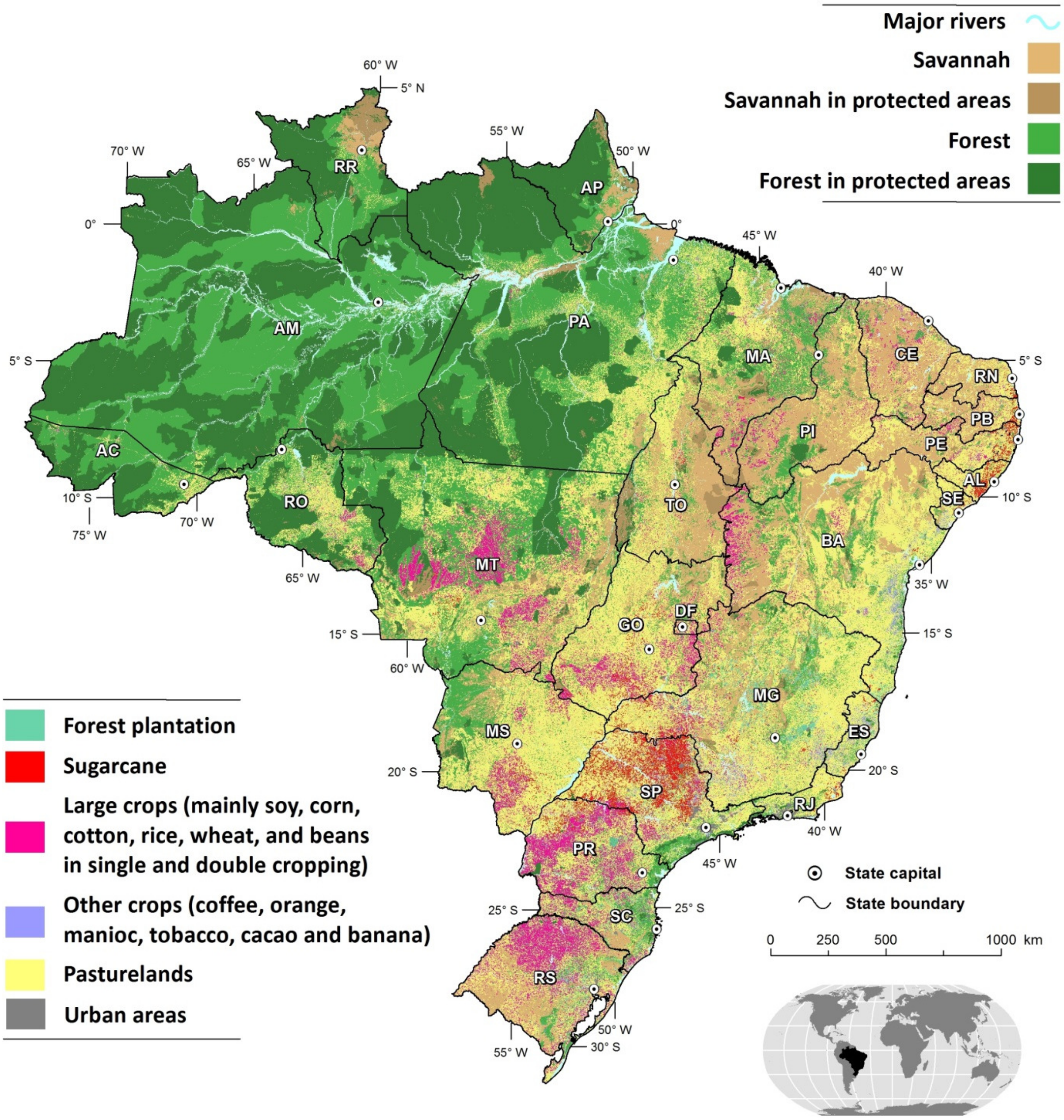

Fig G. Land use in Brazil, as of 2012 [7].

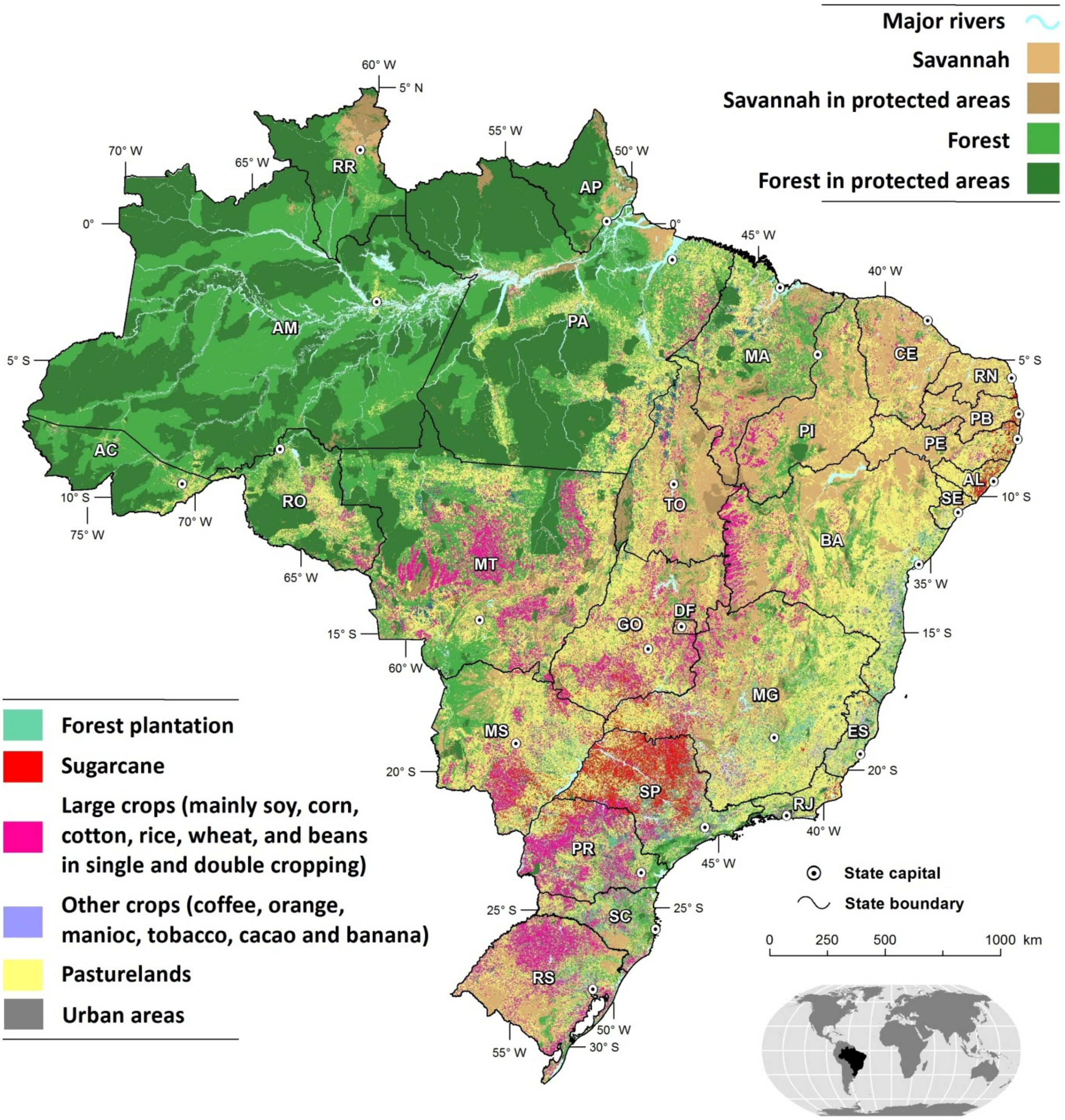

Fig H. Simulated land use in Brazil by 2030 under modeled agricultural expansion scenario.

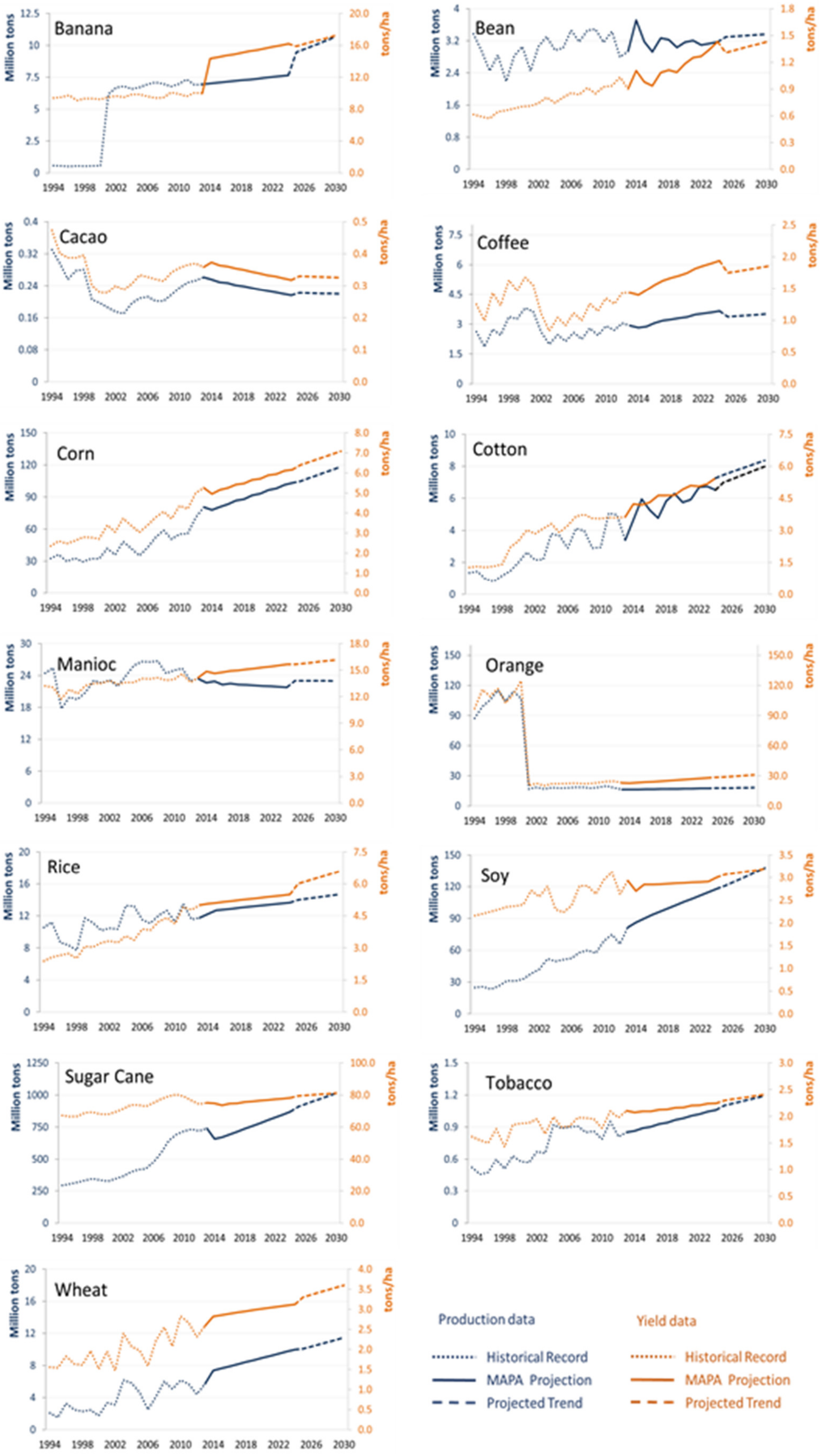

Fig I. Projected yield and production for major crops extrapolated to 2030 by using historical trends between 1994 and 2013 [23] and MAPA projections [25].

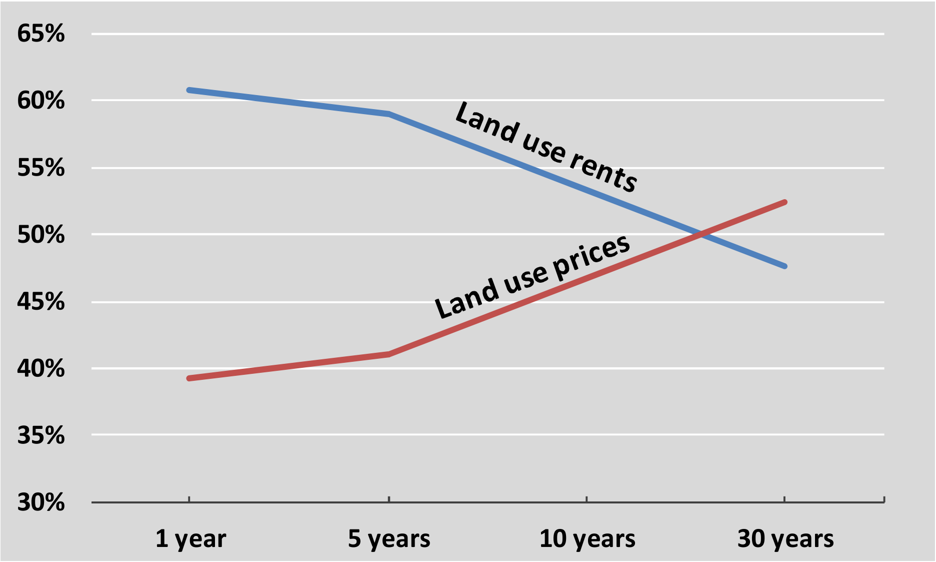

Fig J. Percent of landowners that perceive land prices versus land-use rents as a proxy for the CRA price as a function of the CRA duration period.

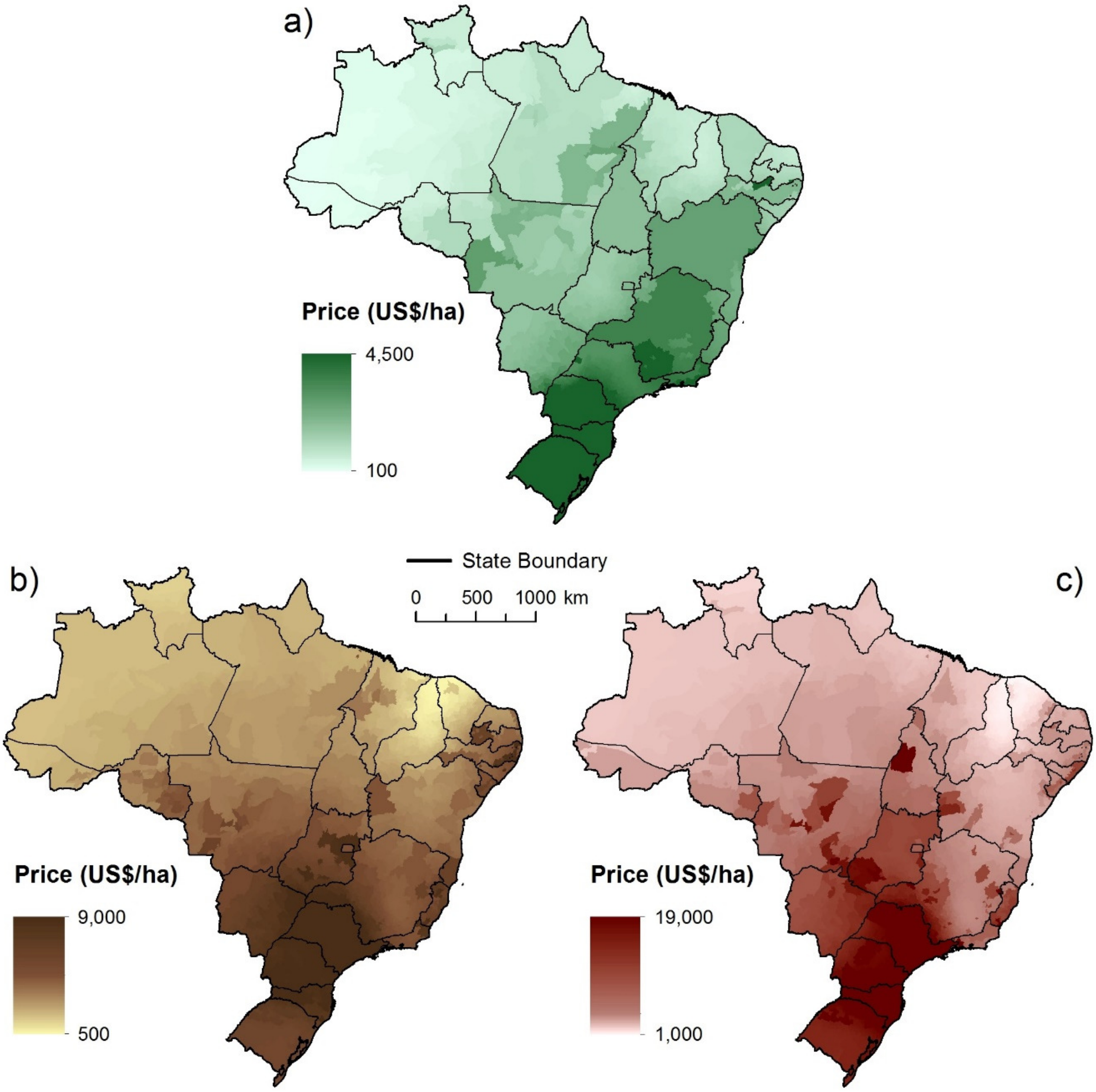

Fig K. Land prices for forested land (a), pastureland (b) and cropland (c) spatially extrapolated from [32].

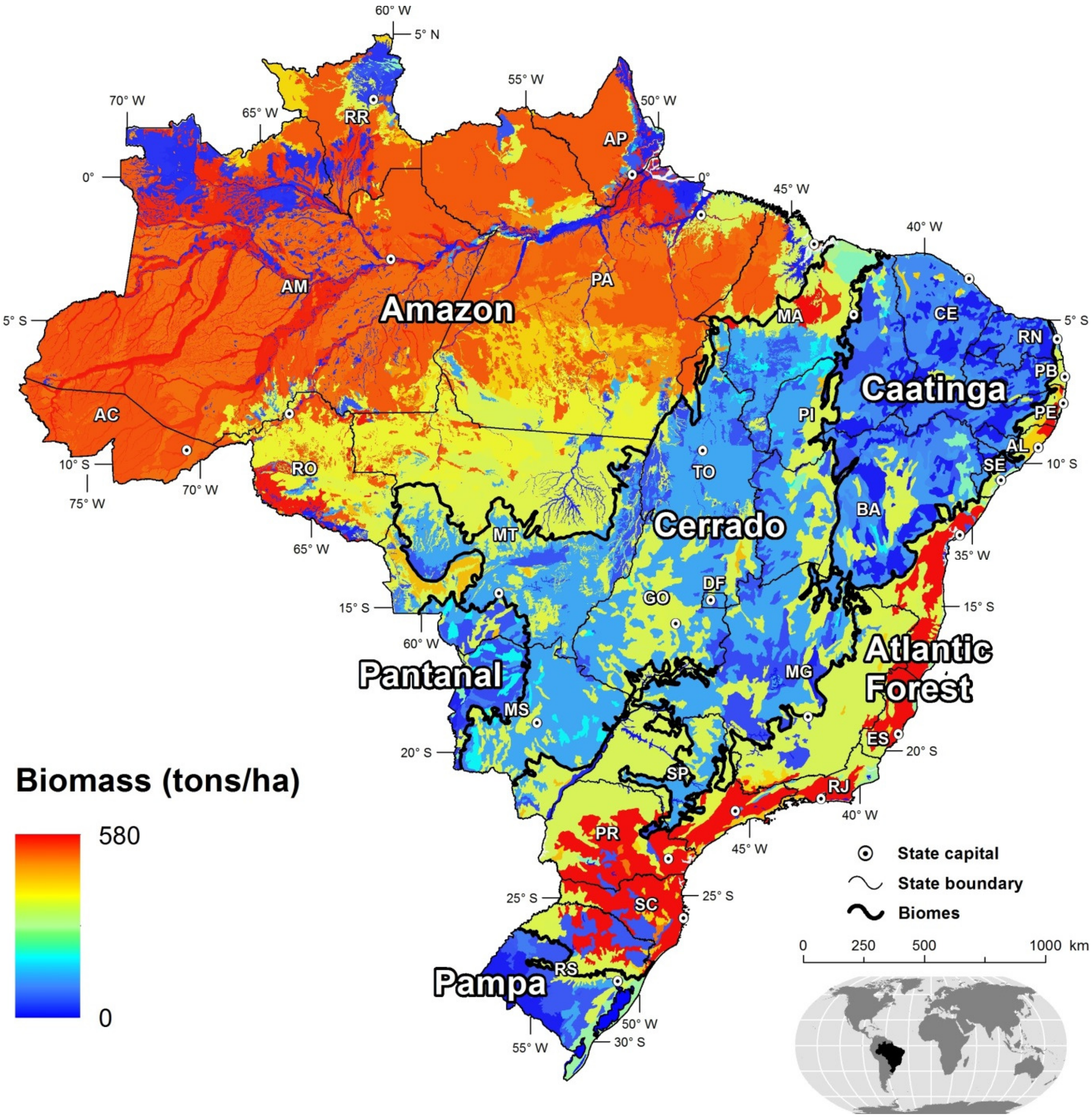

Fig L. Potential above and below ground biomass.

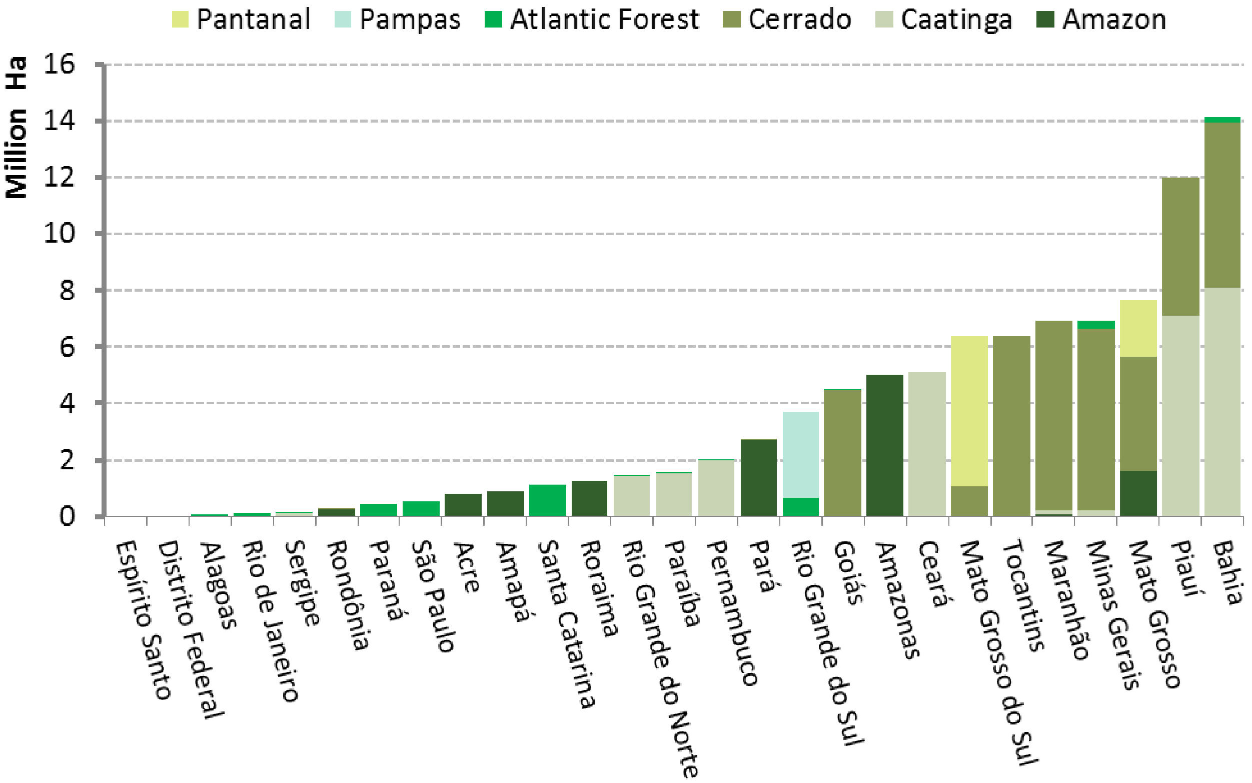

Fig M. FC surplus per state and biome.

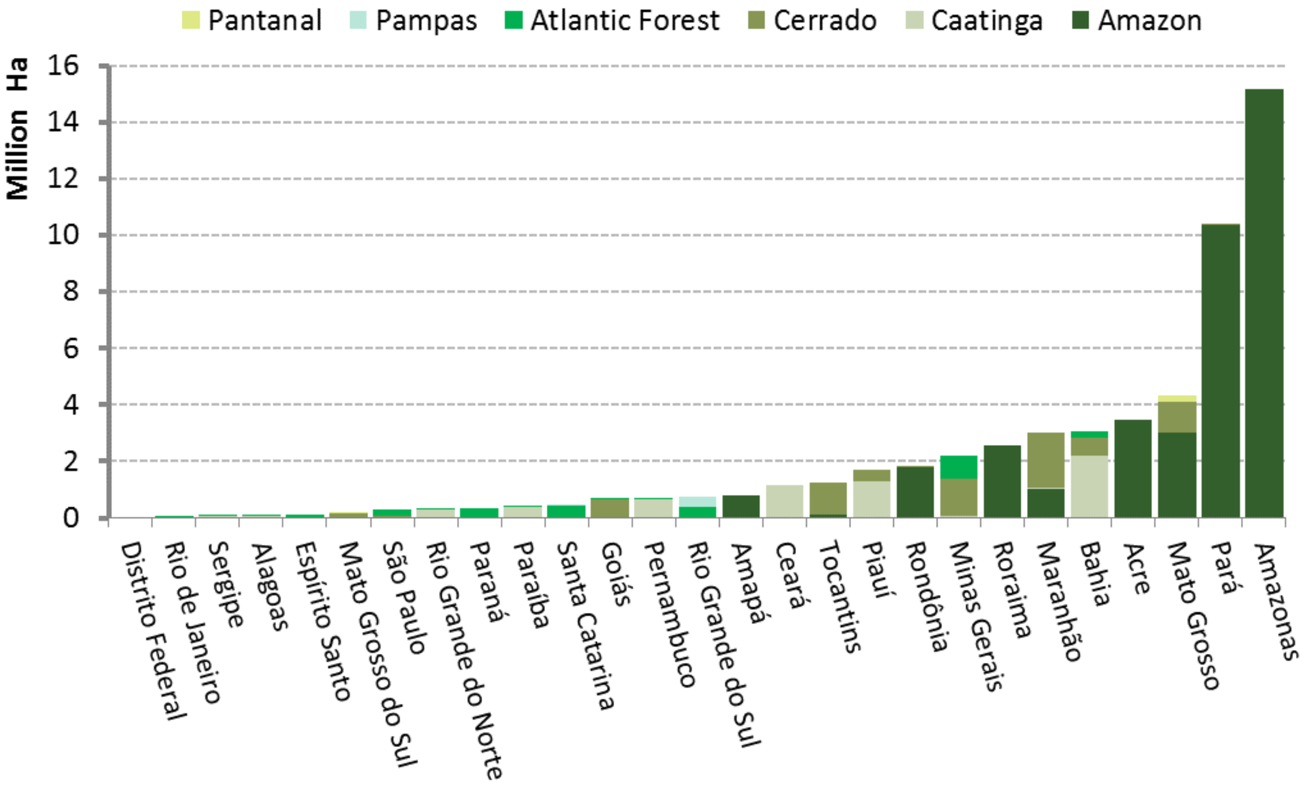

Fig N. LR of small landholders per state and biome.

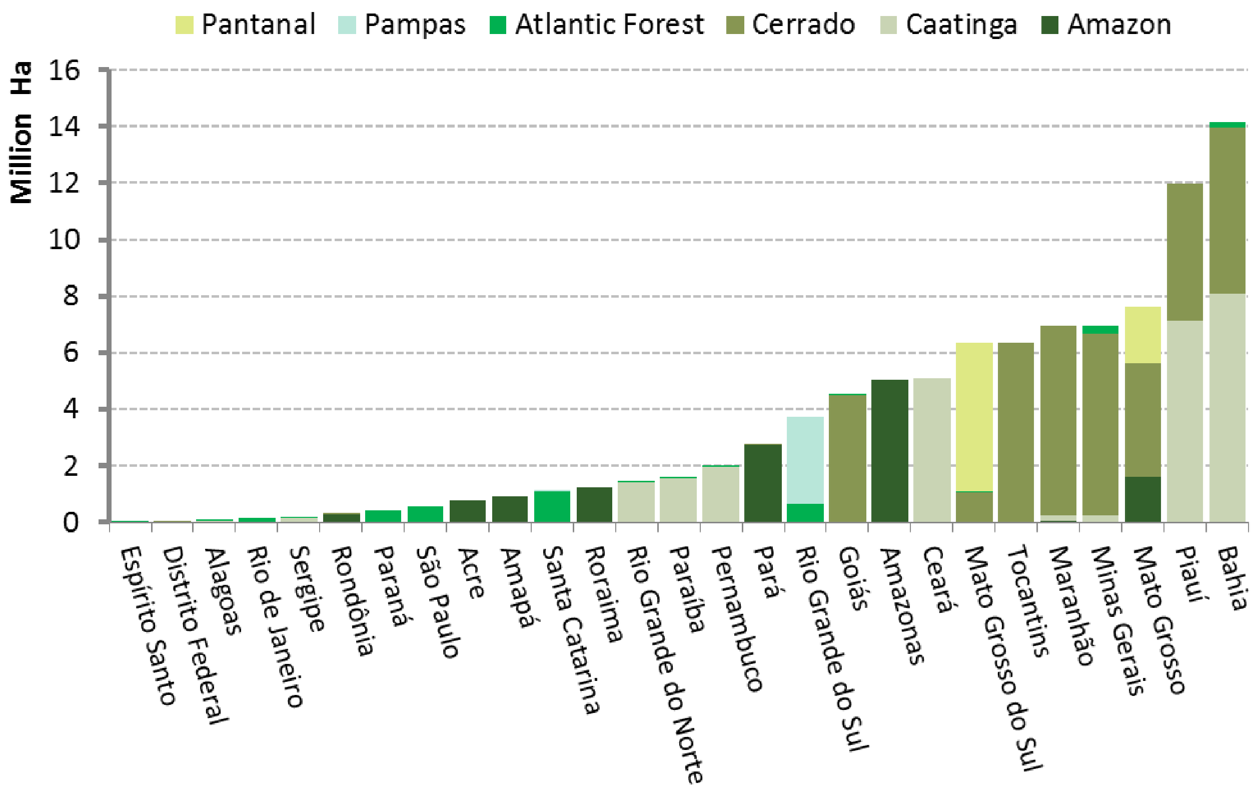

Fig O. LR debts per state and biome.

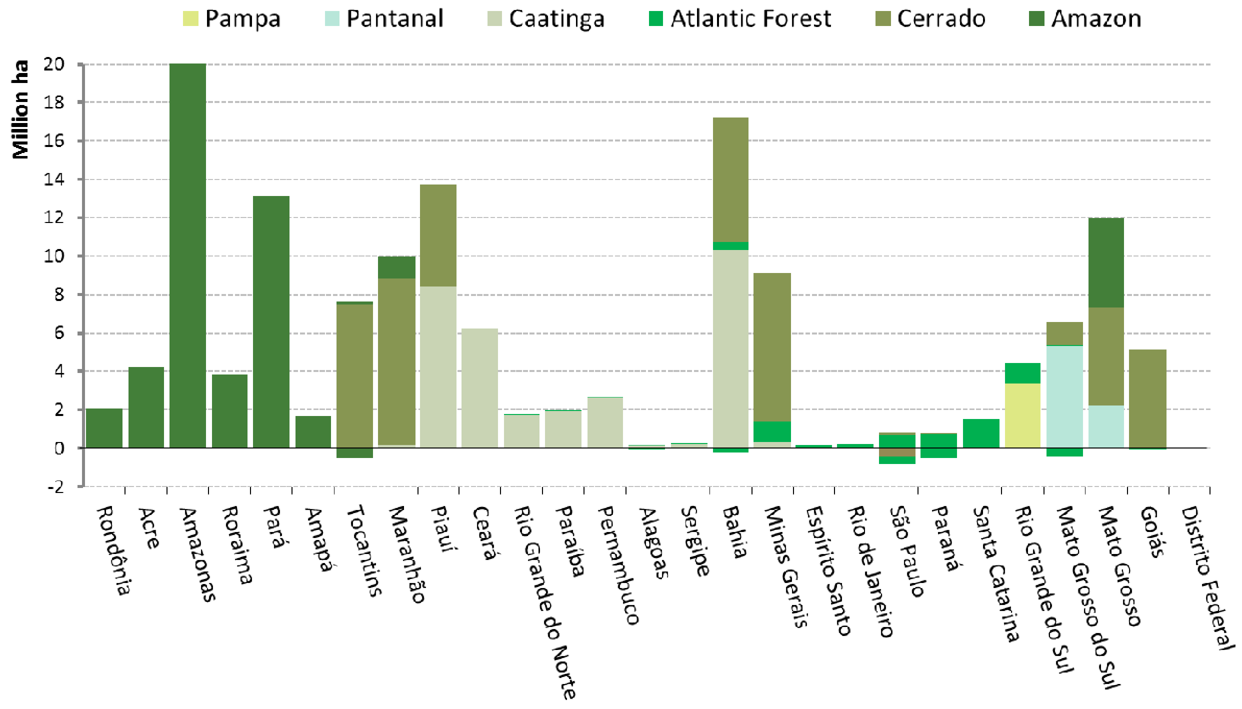

Fig P. Balance between total supplies and LR debts within the same biome and state. Positive numbers represent the potential amount LR that would be offset, while negative numbers depict the remainder LR debts where supplies do not meet regional demands.

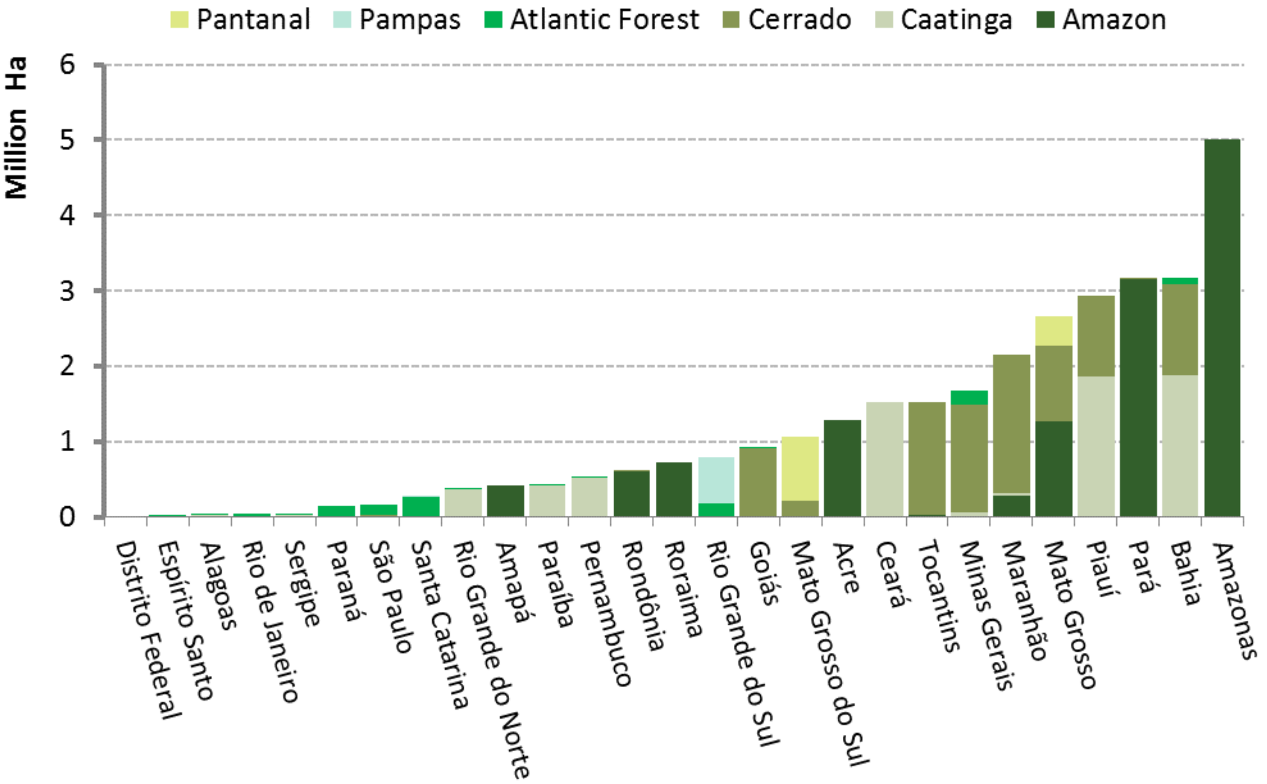

Fig Q. Land without clear land titling per biome and state.

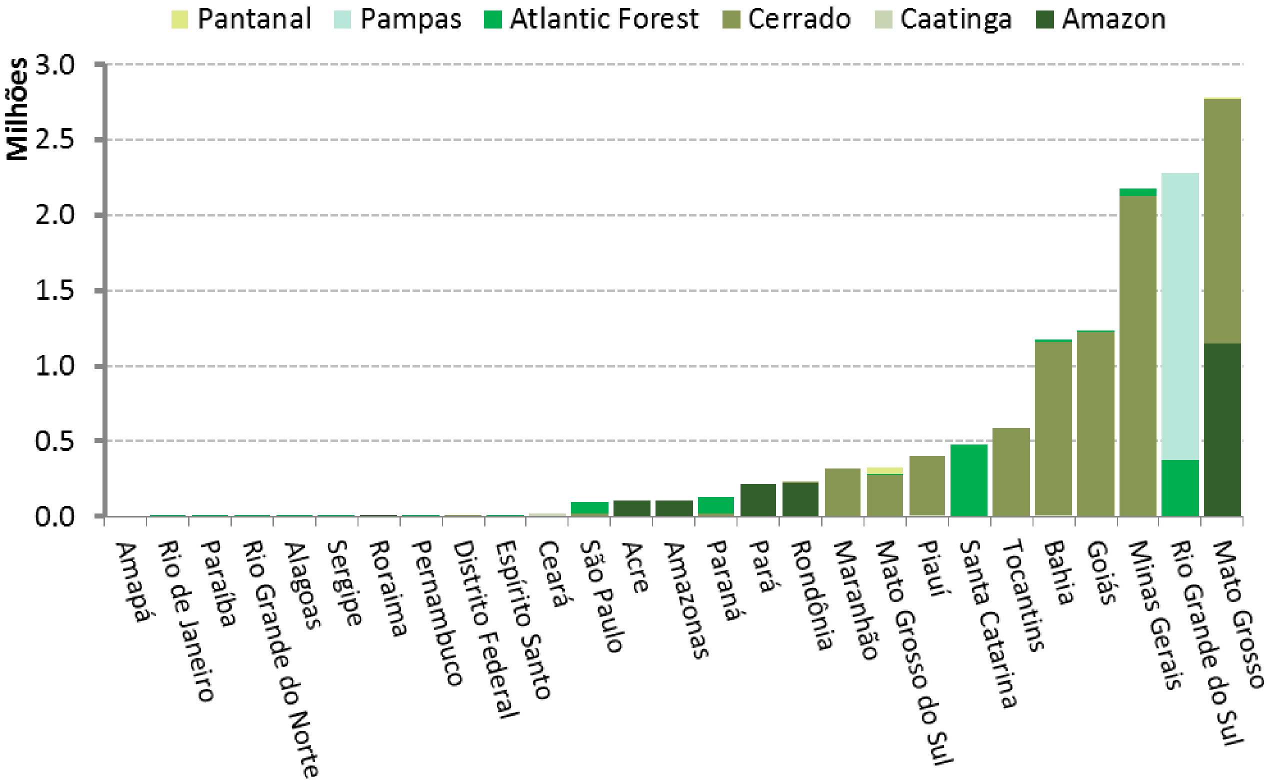

Fig R. Potential CRA forest areas suitable for mechanized agriculture per biome and state.

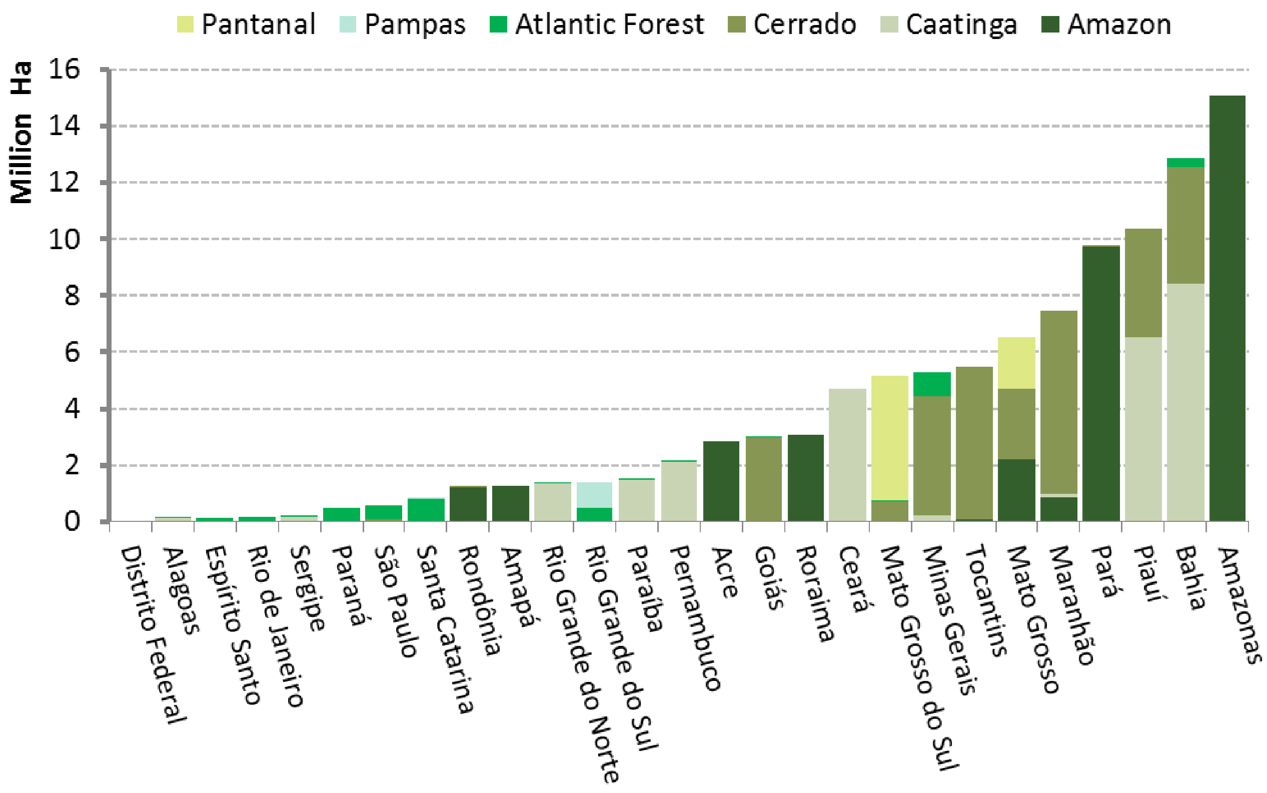

Fig S. Potential supply of CRAs per biome and state.

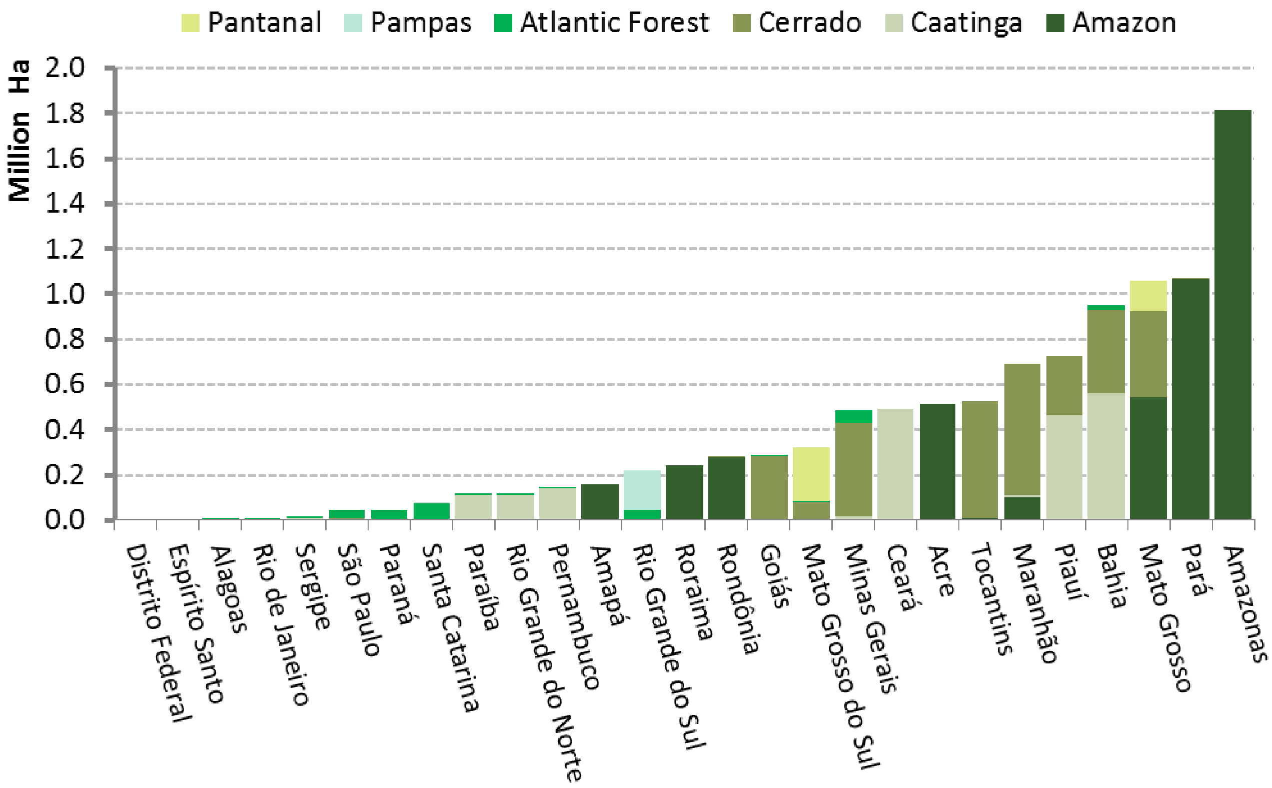

Fig T. CRAs from settlement projects per biome and state.

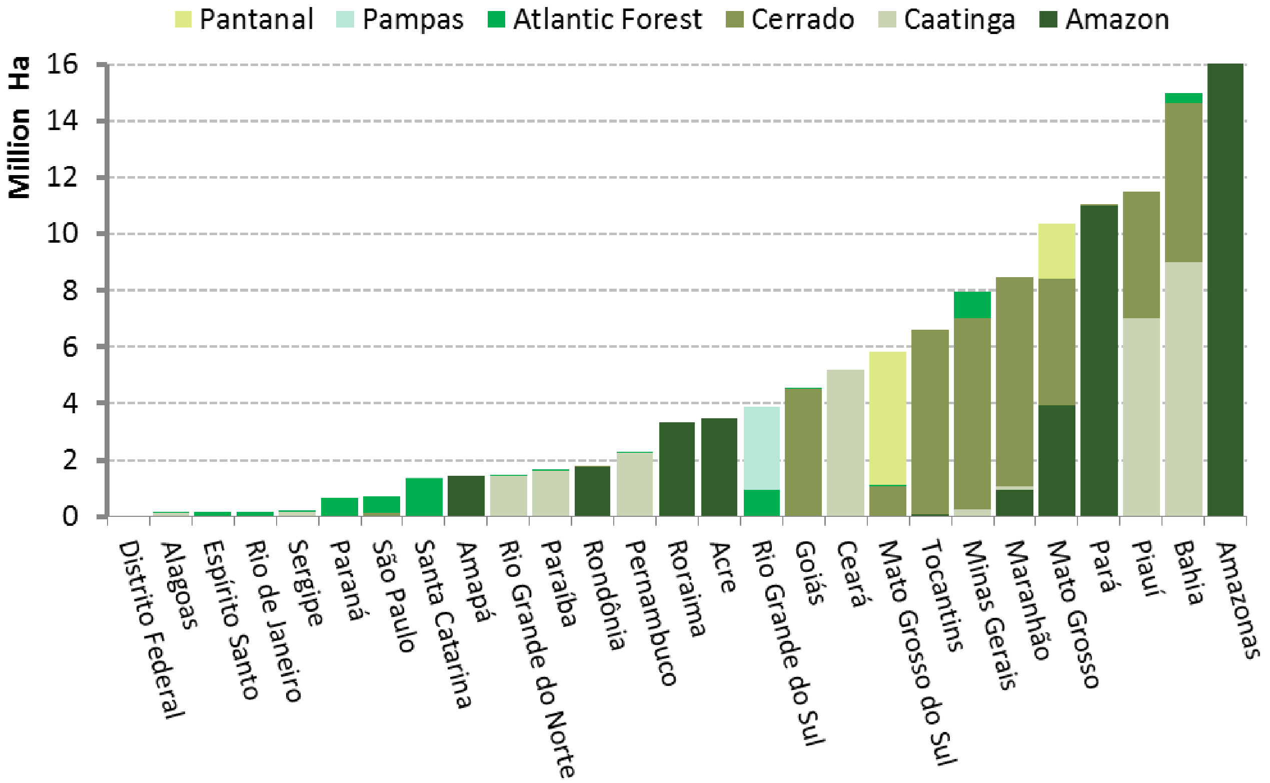

Fig U. Expanded supply of CRAs per biome and state.

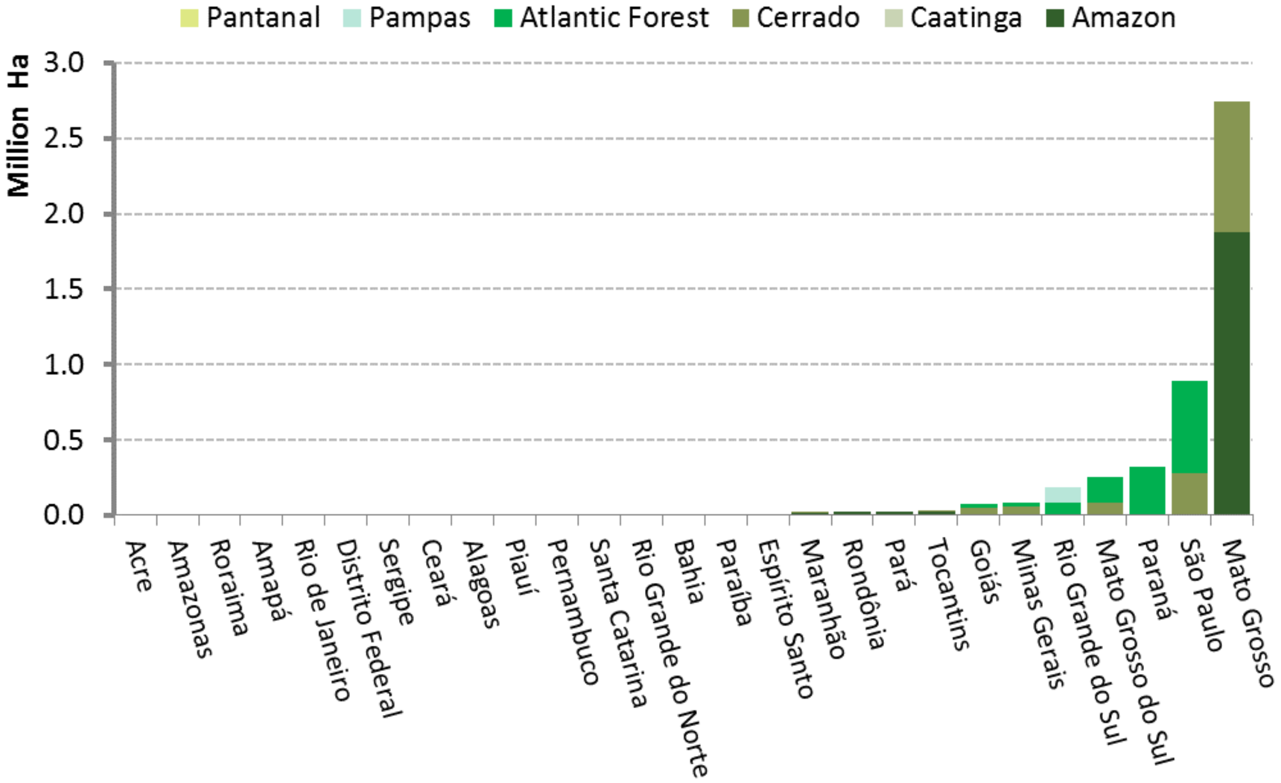

Fig V. Effective demand of CRA certificates by landowners with high land-use opportunity cost per biome and state.

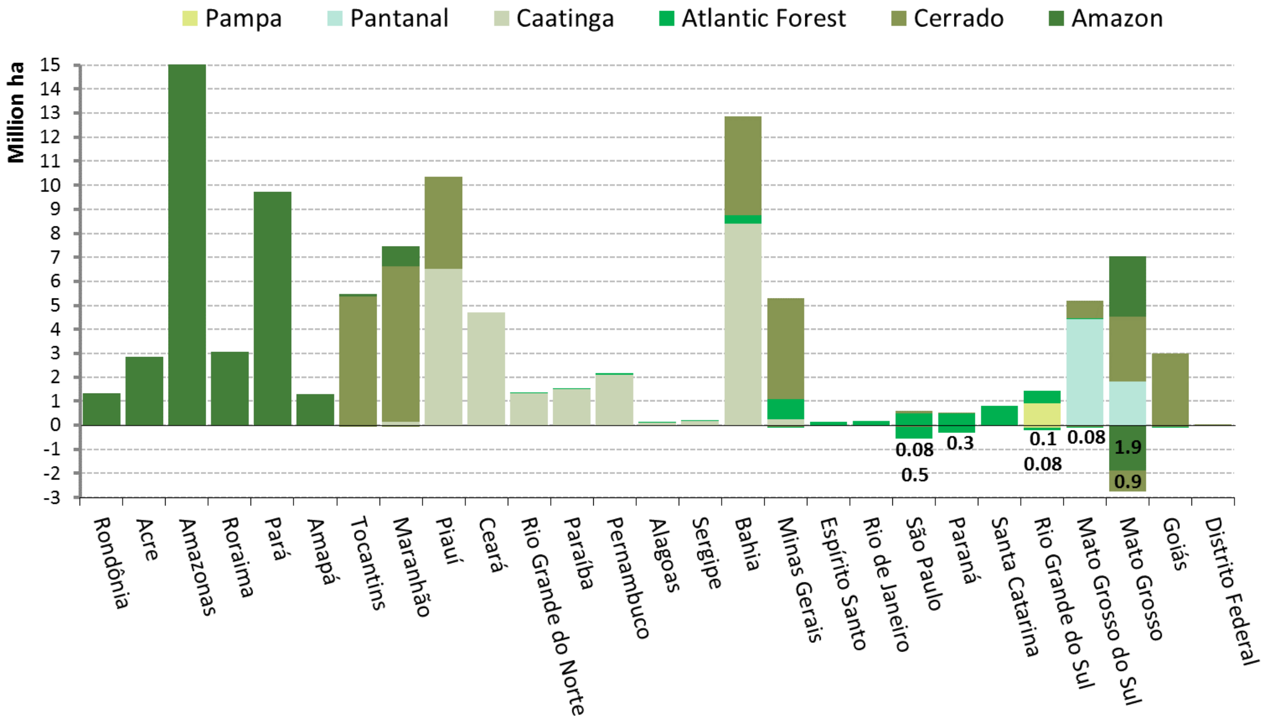

Fig W. Balance between supply (positive numbers) and demand (negative) of CRAs within the same biome and state in Scenario 1. Promising regional markets are highlighted with their potential CRA trading volumes in ha.

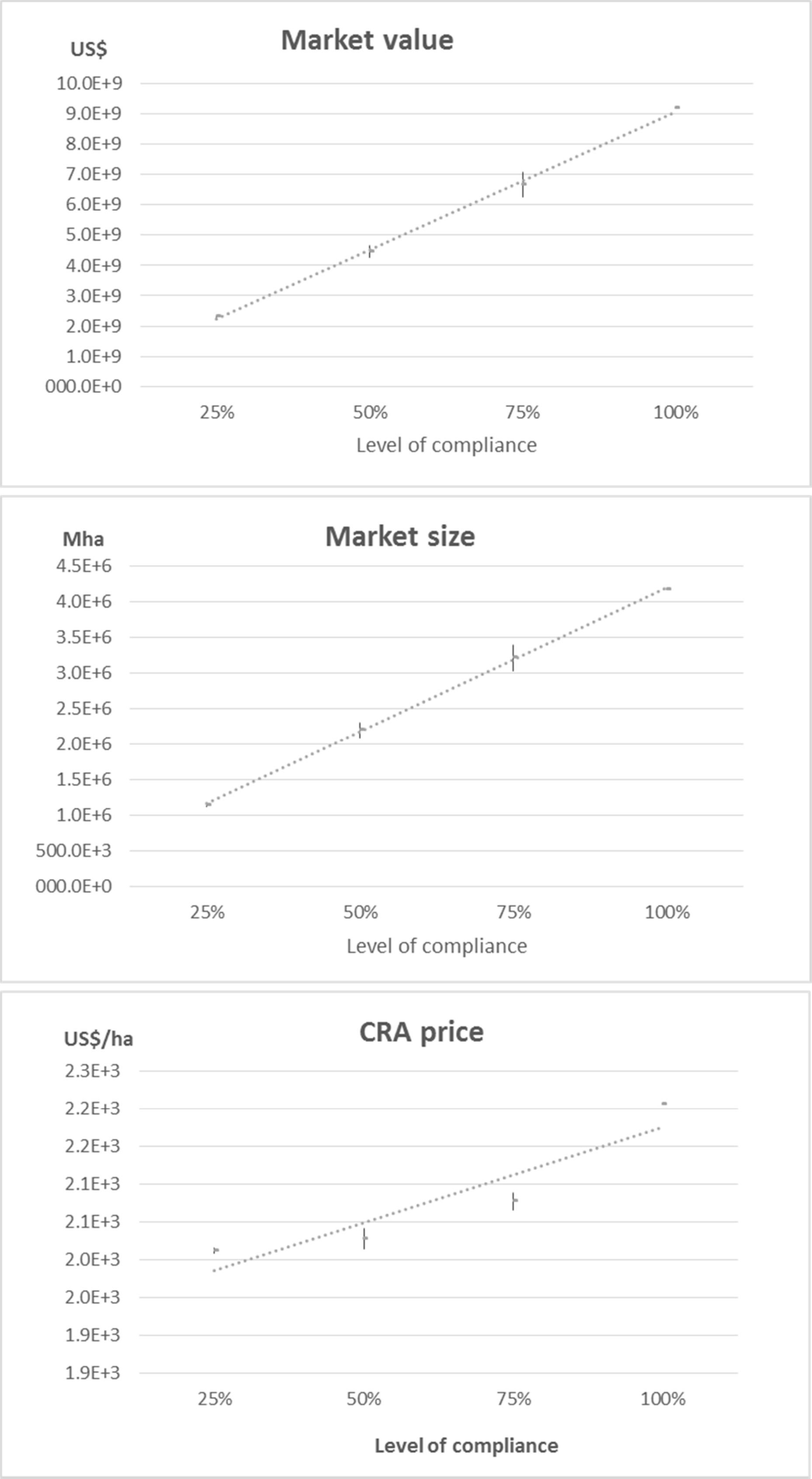

Fig X. Sensitivity analysis results for market value and size and CRA price. Vertical bars represent output intervals from 10 model runs. Dashed lines represents the reduction trends based on the average values (horizontal trace).

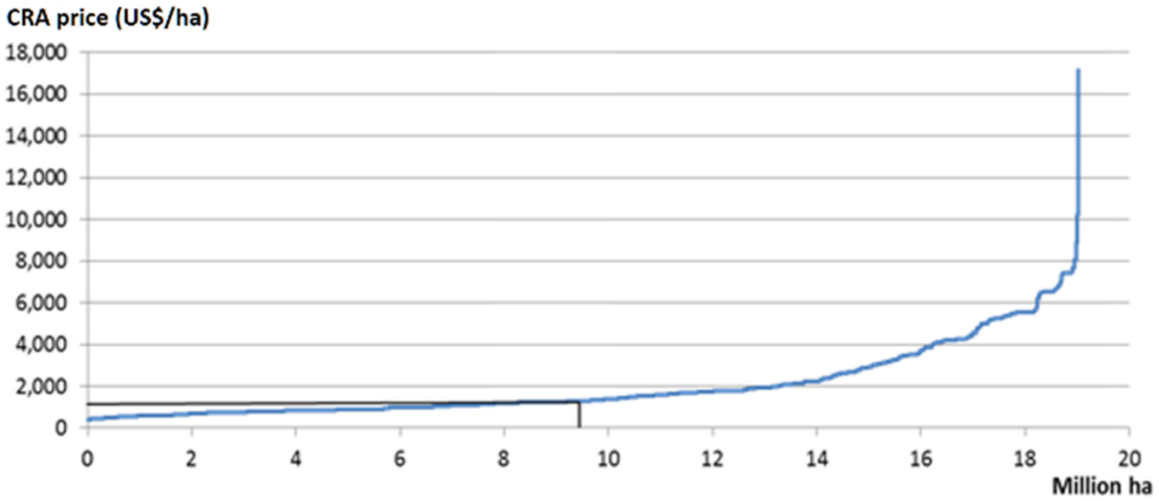

Fig Y. Abatement curve for reducing legal deforestation by 2030 via XCRA market. The black lines indicate the volume and top price of CRAs to be purchased to reduce deforestation by 50%.

SI Tables

Table A. Total of CRAs from conservation units.

Biome	Volume (Ha)	Mean price (US$/ha)
Amazon	14,069,498	702
Caatinga	253,059	1,016
Cerrado	2,031,442	1,047
Atlantic Forest	559,395	2,270
Pampa	0	2,115
Pantanal	0	1,347

Access S2 File for information on inholdings.

Table B. Areas of major crops by 2030 under the modeled agricultural scenario. Total area does not include double cropping.

Crop/plantation	Mha
Soya beans	43.1
Sugar cane	12.5
Corn	6.7
Cotton	1.3
Rice	2.2
Beans	1.2
Arabica Coffee	1.4
Robusta Coffee	0.5
Orange	0.6
Manioc	1.4
Banana	0.6
Cocoa	0.7
Tobacco	0.5
Forest plantation	10.5
Corn double cropping	10.0
Wheat double cropping	3.2
Bean double cropping	1.2
**Total Area**	**83.1**

Table C. Mean historical deforestation rates for Brazilian biomes.

Biome	mean annual deforestation in km^2	Time-period	source
Amazon	5,746	2009-2014	INPE (10)
Caatinga	1,430	2008-2013	Hansen et al. (12)
Cerrado	5,343	2009-2014	LAPIG (20)
Atlantic Forest	188	2009-2014	SOSMA (11)
Pampa	222	2008-2013	Hansen et al. (12)
Pantanal	305	2008-2013	Hansen et al. (12)

Table D. Cost of fencing. Parcel size refers to number of ha of CRAs traded all together.

Cost of fencing per parcel size of CRAs	Fence perimeter (m)	US$	US$/ha
0 ≤ 20 ha	949	3,380	338
20 ≤50 ha	1,775	6,323	181
50 ≤100 ha	2,598	9,256	123
100 ≤200 ha	3,674	13,091	87
200 ≤ 500 ha	5,612	19,996	57
500 ≤1000 ha	8,216	29,271	39
1000 ≤2500 ha	12,550	44,713	26
> 2500 ha	18,371	65,453	17

Table E. Mean transaction costs (US$/ha) per parcel size of CRAs.

Transaction cost per parcel size of CRAs	30-year CRA	5-year CRA
0 ≤ 20 ha	200±100	200±100
20 ≤50 ha	96±66.3	57±28.5
50 ≤100 ha	45±30.3	27±12.9
100 ≤200 ha	25±13.2	14±6.2
200 ≤ 500 ha	13±2.7	6±1.6
500 ≤1000 ha	7±0.7	3±0.7
1000 ≤2500 ha	3±0.3	1±0.3
> 2500 ha	1±0.1	1±0.1

Table F. List of authors who provided additional information about published work and/or unpublished data.

Authors who provided additional information	Additional details	Unpublished data	Reference
Letícia de Barros Viana Hissa	Yes	No	Leite et al. (39)
Alexander C. Vibrans, Débora V. Lingner	Yes	Yes	Vibrans et al. (40)
Milena Rosenfield	Yes	Yes	Rosenfield & Souza (41)
Fabrício Terra	No	Yes	Terra et al. (42)
Tânia Lucia da Costa	Yes	Yes	Costa et al. (43)
Edward Mitchard	Yes	No	Mitchard et al. (44)
Gabriela Lopez-Gonzalez	Yes	No	Lopez-Gonzalez et al. (45)
Paulo M. de Alencastro Graça	Yes	No	Graça et al. (46)
Heloísa Sinátora Miranda	Yes	No	Ottmar et al. (47)
Everardo V. de Sá Barretto Sampaio	Yes	No	Sampaio & Costa (48)

Table G. Traded areas and economic data output from the model for each state and biome unit under the regulatory scenario 1.

State	Biome	Traded area (Ha)	Market value (US$)	Equilibrium price (US$/ha)
Rondônia	Amazônia	22,168	24,023,926	1,084±279
Pará	Amazônia	22,795	13,390,737	587±38
Tocantins	Amazônia	24,455	23,567,795	964±135
Tocantins	Cerrado	10,685	7,842,293	734±74
Maranhão	Amazônia	17,898	13,419,338	750±134
Maranhão	Cerrado	61	28,769	472±61
Mato Grosso	Amazônia	1,880,096	2,705,589,313	1,439±315
Mato Grosso	Cerrado	864,466	1,237,593,650	1,432±394
Ceará	Caatinga	138	66,753	484±31
Rio Grande do Norte	Caatinga	913	687,086	753±218
Rio Grande do Norte	Mata Atlântica	1,781	1,886,203	1,059±376
Paraíba	Caatinga	379	458,326	1,209±322
Paraíba	Mata Atlântica	4,083	7,229,759	1,771±557
Pernambuco	Caatinga	766	823,005	1,074±151
Pernambuco	Mata Atlântica	116	186,679	1,609±506
Bahia	Caatinga	225	253,988	1,129±130
Bahia	Cerrado	625	729,942	1,168±191
Bahia	Mata Atlântica	3,074	3,847,504	1,252±252
Piauí	Cerrado	498	236,724	475±64
Minas Gerais	Cerrado	56,561	77,475,416	1,370±305
Minas Gerais	Mata Atlântica	27,764	46,372,337	1,670±333
São Paulo	Cerrado	75,253	450,117,944	5,981±2,310
São Paulo	Mata Atlântica	488,471	2,696,587,116	5,520±1,773
Paraná	Cerrado	72	225,008	3,125±671
Paraná	Mata Atlântica	318,666	1,117,554,251	3,507±747
Mato Grosso do Sul	Cerrado	81,649	155,209,053	1,901±672
Mato Grosso do Sul	Mata Atlântica	13,621	36,732,353	2,697±897
Goiás	Cerrado	51,724	63,560,376	1,229±318
Goiás	Mata Atlântica	4,374	11,632,806	2,660±902
Alagoas	Mata Atlântica	457	744,166	1,628±585
Sergipe	Mata Atlântica	104	151,888	1,460±482
Espírito Santo	Mata Atlântica	5,940	12,031,953	2,026±588
Santa Catarina	Mata Atlântica	1,289	3,432,937	2,663±577
Rio Grande do Sul	Mata Atlântica	83,041	223,708,592	2,694±478
Rio Grande do Sul	Pampas	107,573	257,674,860	2,395±332
**Totals and mean price**		**4,171,781**	**9,195,072,846**	**2,204±581**
